# Neonatal corticosterone mitigates autoimmune neuropsychiatric disorders associated with streptococcus in mice

**DOI:** 10.1038/s41598-018-28372-3

**Published:** 2018-07-05

**Authors:** Simone Macrì, Chiara Spinello, Joanna Widomska, Roberta Magliozzi, Geert Poelmans, Roberto William Invernizzi, Roberta Creti, Veit Roessner, Erika Bartolini, Immaculada Margarit, Jeffrey Glennon, Giovanni Laviola

**Affiliations:** 1 Centre for Behavioural Sciences and Mental Health, Istituto Superiore di Sanità, Roma, Italy; 2 Department of Cognitive Neuroscience, Donders Institute for Brain, Cognition and Behaviour, Radboud University Medical Center, Nijmegen, The Netherlands; 3 Department of Human Genetics, Radboud University Medical Center, Nijmegen, The Netherlands; 4 Neurology B, Department of Neurological, Biomedical and Movement Sciences, University of Verona, Verona, Italy; 5 Division of Brain Sciences, Faculty of Medicine, Imperial College London, London, United Kingdom; 6 IRCCS-Istituto di Ricerche Farmacologiche “Mario Negri”, Milano, Italy; 7 Respiratory and Systemic Bacterial Diseases Section, Department of Infectious, Parasitic, and Immune-mediated Diseases, Istituto Superiore di Sanità, Roma, Italy; 8 Department of Child and Adolescent Psychiatry, Faculty of Medicine of the TU Dresden, Dresden, Germany; 9 GSK, Siena, Italy

## Abstract

Increased glucocorticoid concentrations have been shown to favor resilience towards autoimmune phenomena. Here, we addressed whether experimentally induced elevations in circulating glucocorticoids mitigate the abnormalities exhibited by an experimental model of Pediatric Autoimmune Neuropsychiatric Disorders Associated with Streptococcus (PANDAS). This is a pathogenic hypothesis linking repeated exposures to Group-A-beta-hemolytic streptococcus (GAS), autoantibodies targeting selected brain nuclei and neurobehavioral abnormalities. To persistently elevate glucocorticoid concentrations, we supplemented lactating SJL/J mice with corticosterone (CORT; 80 mg/L) in the drinking water. Starting in adolescence (postnatal day 28), developing offspring were exposed to four injections - at bi-weekly intervals - of a GAS homogenate and tested for behavioral, immunological, neurochemical and molecular alterations. GAS mice showed increased perseverative behavior, impaired sensorimotor gating, reduced reactivity to a serotonergic agonist and inflammatory infiltrates in the anterior diencephalon. Neonatal CORT persistently increased circulating glucocorticoids concentrations and counteracted these alterations. Additionally, neonatal CORT increased peripheral and CNS concentrations of the anti-inflammatory cytokine IL-9. Further, upstream regulator analysis of differentially expressed genes in the striatum showed that the regulatory effect of estradiol is inhibited in GAS-treated mice and activated in GAS-treated mice exposed to CORT. These data support the hypothesis that elevations in glucocorticoids may promote central immunomodulatory processes.

## Introduction

Neonatal experiences persistently adjust individual adaptation towards future challenges^1^. While adverse events may relate to an increased risk of pathology^2^, stimulating neonatal conditions may favor resilience towards future environmental insults^3^. Although these considerations originally pertained to emotional^4^ and cognitive^5^ domains, they have been recently extended to the immune system. For example, Bakker and collaborators^6^ observed that neonatal dexamethasone administration – exerting a long-term reduction of corticosterone reactivity – increased individual susceptibility to an experimental autoimmune disease in rats. Meagher *et al*.^7^ reported that brief (15-min/day) and long (180-min/day) maternal separations during the first two weeks of life increased vulnerability to acute Theiler’s virus infection^7^. Importantly, also in this study, exposure to neonatal stressors resulted in a blunted activity of the hypothalamic-pituitary-adrenocortical (HPA) axis. These data support the hypothesis that experimental interventions capable of reducing corticosterone reactivity to stressors may potentiate autoimmune responses^8^. Likewise, experimentally-induced increases in corticosterone reactivity have been reported to favor resilience towards autoimmunity in experimental allergic encephalomyelitis (EAE). Specifically, Levine and collaborators exemplified the bimodal regulatory role of corticosteroids on individual reactivity to autoimmunity by showing that stress suppresses^9^ and adrenalectomy potentiates^10^ vulnerability to EAE.

Epidemiological, clinical and preclinical data support the notion that autoimmune phenomena constitute a vulnerability factor in the onset of psychiatric disturbances. Swedo and colleagues coined the acronym PANDAS (Pediatric Autoimmune Neuropsychiatric Disorders Associated with Streptococcus) to define a series of motor disturbances in which repeated exposures to bacterial (Group-A β-Hemolytic Streptococcus, GAS) infections are causally linked to symptoms^11^. Besides its pediatric onset, the defining criteria of PANDAS include the temporal proximity of neurological disturbances and GAS infections, recurrent abnormal behaviors, and remitting-relapsing presence of obsessive-compulsive symptoms and/or tics^11^. A proof of principle of these pathological sequelae has been obtained through several independent preclinical studies^12–14^. Hoffman *et al*. observed that repeated exposures to GAS homogenate resulted in locomotor alterations associated with increased IgG antibody deposits in deep cerebellar nuclei. We recently reported that in the long term, an analogous treatment resulted in increased repetitive behaviors, impaired sensorimotor gating, and inflammatory processes in the rostral diencephalon^14^. Tourette’s Syndrome (TS) and Obsessive Compulsive Disorder (OCD) have been proposed to constitute instances of PANDAS: while several studies reported the presence of elevated concentrations of anti-streptococcal antibody titers in TS patients^15^, Orlovska *et al*.^16^ demonstrated that streptococcal infections relate to an increased risk of OCD in a Danish nationwide study. Both TS and OCD have been associated with alterations at the level of the basal ganglia^17^.

**Figure 1:**
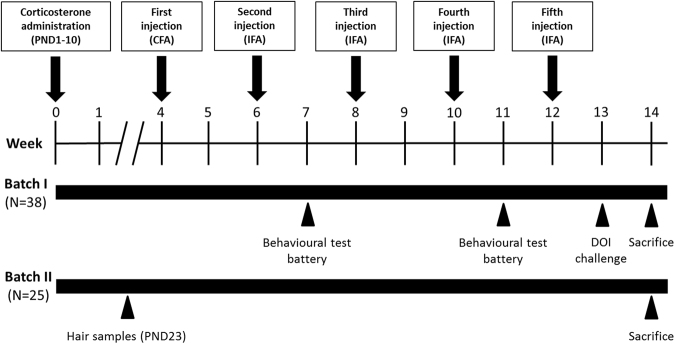
Experimental design: Timing of the neonatal treatment and of the injections, expressed in weeks, and the experimental procedures performed with two different and independent batches of mice. After corticosterone administration during the neonatal phase (postnatal days, PND, 1–10), mice received 5 injections of GAS homogenate or Phosphate Buffer Saline (PBS), formulated with the indicated adjuvants (CFA = Complete Freund’s adjuvant; IFA = Incomplete Freund’s adjuvant). At sacrifice, we collected: in Batch I, plasma samples for antibody and cytokine determination and brain samples for immunohistochemistry; in Batch II, we collected brain areas for monoamine determinations and RNA sequencing.

Here, we addressed through a longitudinal study (see Fig. 1 for the study outline) conducted in mice whether neonatal stress may modulate individual susceptibility to PANDAS^14^. We thus tested the prediction that neonatal CORT administration – mimicking sustained chronic stress – mitigates behavioral, immunological and neurochemical abnormalities in SJL mice repeatedly exposed to GAS between late infancy and adulthood. To elucidate the molecular mechanisms underlying the observed phenotypic alterations, we performed RNA sequencing of the striatum, the main component of the basal ganglia that is typically affected in PANDAS^17^.

## Results

### Physiological indicators of the efficacy of experimental treatments

#### Neonatal CORT administration increases circulating CORT concentrations in the short- and long-term

**Figure 2:**
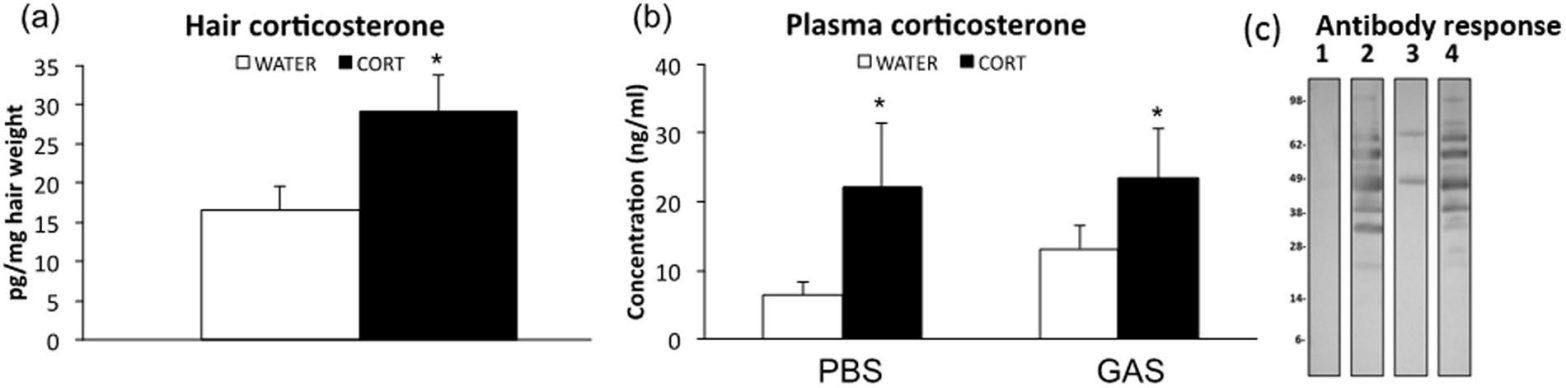
(**a**) Hair corticosterone (CORT) concentrations measured at weaning in control (WATER) and CORT-treated subjects (n = 11 per group); (**b**) Plasma CORT concentrations measured at sacrifice (i.e. two weeks after the fifth GAS injection; n = 8–12 per group): log-transformed data were used for the statistical analysis; actual concentration data are reported for the sake of clarity. CORT-treated subjects showed increased concentrations of CORT both in the short- (weaning) and long-term (adulthood). ^*^p < 0,05 compared to respective WATER controls; (**c**) Western Blot analysis of GAS extracts probed with pooled sera from mice treated with GAS homogenates or adjuvant alone as Control, neonatal CORT and GAS homogenates or neonatal CORT and adjuvant. Lane 1, Western Blot results after probing with sera of control mice injected with four doses of adjuvant alone; lane 2, Western Blot results after probing with sera of mice injected with four doses of GAS homogenates; lane 3, Western Blot results after probing with sera of mice injected with four doses of neonatal CORT and adjuvant; lane 4, Western Blot results after probing with sera of mice injected with four doses of neonatal CORT and GAS homogenates. Lanes have been cropped from different parts of the same gel (see Supplementary Fig. 2).

Hair CORT concentrations at weaning were significantly elevated in CORT offspring compared to WATER controls (neonatal treatment: *F*(1,21) = 4,839, *p* = 0,0392, see Fig. 2a). An analogous increment in CORT concentrations was also observed in adulthood (see Fig. 2b), irrespective of GAS administration, when we evaluated serum basal CORT concentrations (neonatal treatment: *F*(1,32) = 4,611, *p* = 0,0394, see Fig. 2b).

#### Repeated GAS exposures elicit the production of anti-GAS antibodies

Western Blot analyses demonstrate that sera from GAS-treated mice recognized specific GAS proteins and suggest that CORT administration did not influence this response (see Fig. 2c).

### Behavioral testing

#### Neonatal CORT administration compensates for the PANDAS-like behavioral abnormalities induced by repeated GAS exposures

In the absence of major effects of experimental treatments on anxiety-related behaviors, general locomotion, and motor coordination (see Supplementary Results and Supplementary Table 1 for details), repeated GAS exposures exerted long-term effects in PANDAS-relevant phenotypes. Additionally, precocious exposure to CORT hampered the onset of most of these alterations.

**Figure 3:**
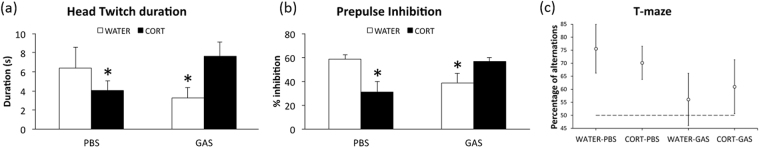
(**a**) Behavioral response (head twitch) to challenge with DOI (5 mg/kg, i.p., week 13, fifth injection) during a single 10-min session. Data are expressed as mean + SEM (n = 8–12 per group); (**b**) Sensorimotor gating measured through PPI (week 11, fourth injection). Average inhibition of the startle reflex to a 120-dB stimulus following the presentation of a pre-pulse of 67, 70, 73, and 76 dB. Values are expressed as mean percentage PPI (%PPI) + SEM (n = 8–12 per group); (**c**) Perseverative behavior in a T-maze (week 11, fourth injection) measured as the percentage of spontaneous alternations (circular symbol) with 95% CI (whiskers). The dashed lines represent chance level; (**a**) CI intersecting the dashed line indicates that percentage of alternations was not statistically different from chance.

To evaluate the functionality of the serotonergic system, we assessed the behavioral response to a pharmacological challenge with the 5-HT2a agonist DOI after the fifth GAS injection (week 13). As expected, administration of DOI resulted in the appearance of *head twitch* in all experimental subjects. While neither neonatal CORT administration nor GAS exposure *per se* apparently influenced duration (neonatal treatment: *F*(1,33) = 0,469, *p* = 0,4981; PBS/GAS treatment: *F*(1,33) = 0,027, *p* = 0,8699), and frequency (neonatal treatment, *F*(1,30) = 0,606, *p* = 0,4424; PBS/GAS treatment, *F*(1,30) = 1,304, *p* = 0,2626) of *head twitch*, individual response to DOI varied depending on early CORT administration and PBS/GAS treatment (*head twitch* duration, neonatal treatment x PBS/GAS treatment: *F*(1,33) = 5,237, *p* = 0,0287; *head twitch* frequency, neonatal treatment x PBS/GAS treatment: *F*(1,30) = 5,311, *p* = 0,0283). Specifically, WATER-GAS mice exhibited reduced *head twitch* compared to WATER-PBS. Furthermore, neonatal CORT administration reduced individual reactivity to DOI in PBS mice. Finally, in accordance with our predictions, neonatal CORT administration prevented the effects of GAS administration (*p* < 0,05 in post hoc tests, see Fig. 3a).

Sensory integration deficits contribute to the PANDAS phenotype. We thus measured sensorimotor gating through PPI after the second (week 7) and fourth injection (week 11). As expected, all subjects showed intact PPI (reduced startle reflex in reaction to prepulse plus pulse trials than pulse alone trials). In accordance with previous evidence indicating that few GAS exposures are insufficient to induce a pathological phenotype, following the second injection, the percentage of inhibition did not vary among groups (neonatal treatment: *F*(1,31) = 2,116, *p = *0,1559; PBS/GAS treatment: *F*(1,31) = 0,061, *p* = 0,8061; neonatal treatment x PBS/GAS treatment, *F*(1,31) = 0,611, *p* = 0,4405). Following the fourth injection, while neither neonatal CORT administration nor GAS exposure *per se* apparently influenced PPI (neonatal treatment: *F*(1,34) = 0,596, *p* = 0,4454; PBS/GAS treatment: *F*(1,34) = 0,208, *p* = 0,6512), individual responses varied depending on the interaction between early CORT administration and PBS/GAS treatment (neonatal treatment x PBS/GAS treatment: *F*(1,34) = 13,256, *p = *0,0009). As expected, GAS administration resulted in impaired PPI in subjects precociously exposed to water. Neonatal CORT administration, which *per se* impaired PPI in PBS mice, prevented the effect of GAS administration (*p* < 0,05 in post-hoc tests, see Fig. 3b).

Finally, to test whether GAS exposures and CORT administration influenced the exhibition of perseverative behavior, we conducted the T-Maze test after the fourth injection (week 11). On average, experimental subjects showed spontaneous alternations, a natural tendency to alternate between the two arms of the apparatus (95% CI 57.15 to 80.35). Such natural tendency, considered an inverse index of perseveration, is variable among experimental conditions. In particular, while mice exposed to CORT showed spontaneous alternations (95% CI 63.90 to 76.53) mice exposed to GAS treatment did not show spontaneous alternation since their preference score did not differ from chance level (95% CI 46.27 to 65.95, see Fig. 3c). Conversely, neonatal corticosterone administration mitigated the consequences of GAS treatment whereby CORT-GAS subjects showed a percentage of spontaneous alternations higher than chance level (95% CI 50.82 to 71.12, see Fig. 3c). Additionally, GAS treatment significantly reduced the percentage of spontaneous alternations compared to WATER-PBS individuals (*F*(1,34) = 9,074, *p* = 0,0049, see Supplementary Fig. 3). With respect to neonatal treatment and its interaction with GAS administration, we did not observe significant differences (neonatal treatment: *F*(1,34) = 0,003, *p* = 0,9559; neonatal treatment x PBS/GAS treatment: *F*(1,34) = 1, 163, *p* = 0,2884).

### Immune activation in response to experimental treatments

**Figure 4:**
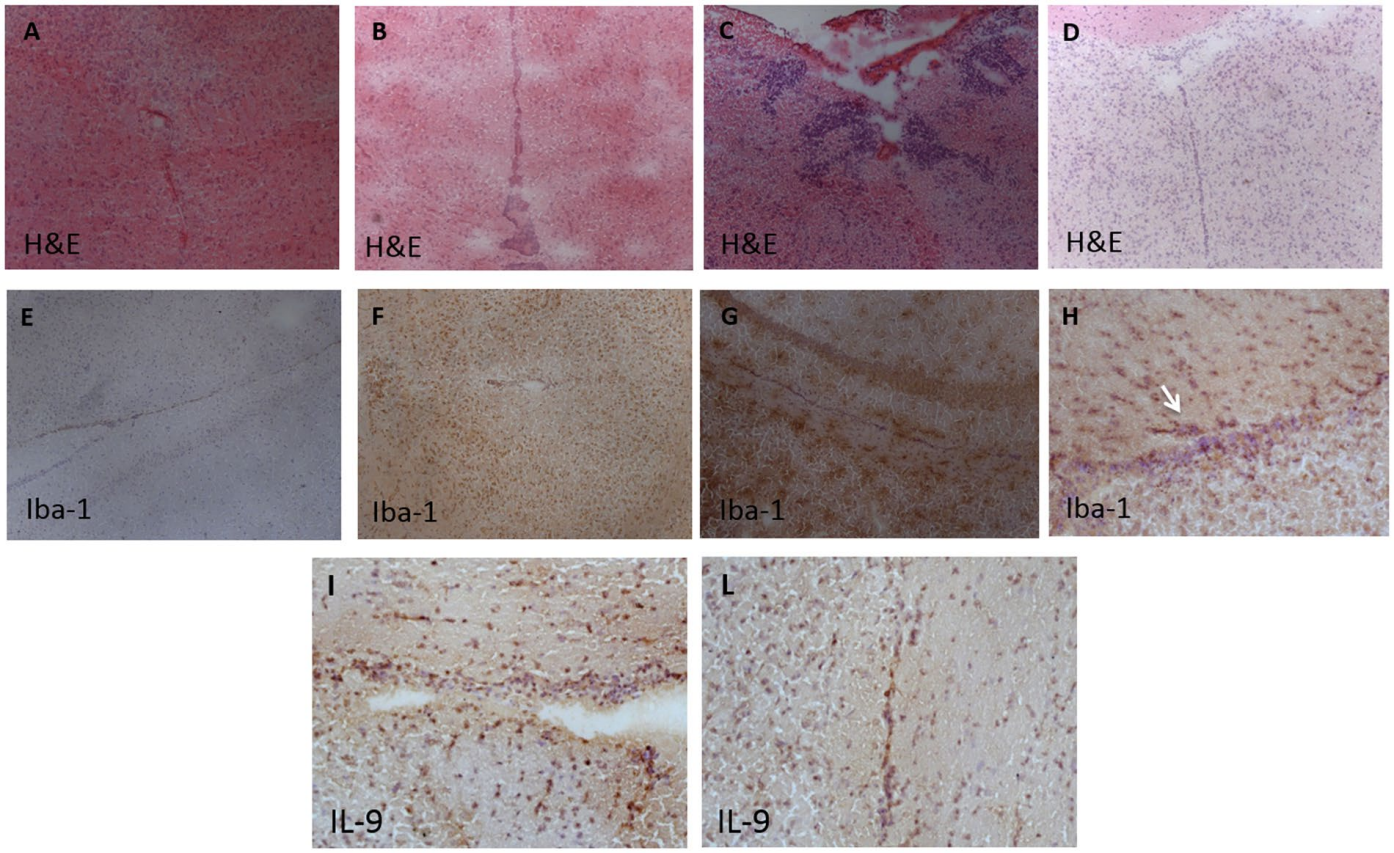
Neuropathology assessment of microglia activity in mice brains. H&E staining shows the presence of inflammatory infiltrates in the white matter of the rostral diencephalon of a representative CORT-PBS mouse (**B**) and a WATER-GAS mouse (**C**) not detected in WATER-PBS mice (**A**) and CORT-GAS mice (**D**). The presence of Iba1+ microglia activity - diffusely present in WATER-GAS mice (**G**)- was rarely observed in CORT-PBS mice (**F**) and CORT-GAS mice (**H**), and was mainly detected associated with small, periventricular inflammatory infiltrates. Iba1+ microglia activity was not detected in WATER-PBS mice (**E**). A considerable number of IL-9+ cells were observed in the inflammatory infiltrates persisting in different regions of the brains of CORT-GAS mice (**I**,**L**). The arrow in H indicates the area in serial brain sections where increased density of IL-9+ cells was detected. (Original magnification: 5x (**B**,**D**), 10x (A,C,E,F,G,L), 20x (**H**,**I**).

We first analyzed whether GAS exposure resulted in inflammatory processes in the brain and then whether CORT compensated for them. In accordance with our predictions, while GAS injections resulted in visible inflammatory infiltrates in adult subjects, neonatal CORT exposure remarkably mitigated this effect (Fig. 4). While CORT-PBS mice and WATER-GAS mice were characterized by the presence of inflammation (Fig. 4B,C respectively), WATER-PBS mice and CORT-GAS mice did not show inflammatory infiltrates (Fig. 4A,D respectively). Furthermore, the diffused microglial activity observed in the mesencephalon of WATER-GAS mice (Fig. 4G) was remarkably reduced in CORT-GAS mice (Fig. 4H), and CORT-PBS mice (Fig. 4F), while was absent in CORT-PBS mice (Fig. 4E). When we analyzed cytokine activation in the plasma (see Supplementary Table 3 for data on cytokine concentrations), we observed that, in the absence of effects due to GAS exposure, CORT administration resulted in remarkable increases of IL-9, IL-17, MCP-1, and MIP-1beta (see Supplementary Table 3 for details). We therefore evaluated whether inflammatory infiltrates were positive for IL-9, a factor indicative of inflammatory processes. Accordingly, we observed a considerable number of IL-9+ cells in the inflammatory infiltrates of CORT-GAS (Fig. 4I,L) but not of CORT-PBS mice.

### Monoamine measurements

Concentrations of serotonin, dopamine, their metabolites and respective turnovers are reported in Supplementary Table 2. In the absence of differences in hippocampus and prefrontal cortex, experimental treatments modulated monoamine concentrations in striatum and hypothalamus. Both CORT and GAS reduced striatal concentrations of DOPAC (neonatal treatment: *F*(1,17) = 12,073, *p* = 0,0029; PBS/GAS treatment: *F*(1,17) = 4,456, *p = *0,0499) and 5HIAA (neonatal treatment: *F*(1,17) = 9,370, *p = *0,0071; PBS/GAS treatment: *F*(1,17) = 6,068, *p* = 0,0247). Furthermore, neonatal CORT administration reduced absolute concentrations of 5-HT in striatum, although this effect was limited to PBS subjects (neonatal treatment: *F*(1,17) = 7,239, *p* = 0,0155, *p* < 0,05 in post-hoc tests). With respect to hypothalamus, GAS administration increased dopamine concentrations only in mice precociously exposed to water and not to CORT (neonatal treatment x PBS/GAS treatment: *F*(1,20) = 8,394, *p* = 0,0089; *p* < 0,05 in post-hoc tests). Furthermore, CORT treatment significantly reduced dopamine turnover (neonatal treatment: *F*(1,20) = 5,184, *p* = 0,0339; WATER-PBS = 1,03 ± 0,17, WATER-GAS = 0,63 ± 0,04, CORT-PBS = 0,62 ± 0,03, CORT-GAS = 0,69 ± 0,08) and serotonin turnover (neonatal treatment: *F*(1,20) = 6,399, *p* = 0,0199; WATER-PBS = 0,85 ± 0,05, WATER-GAS = 0,68 ± 0,06, CORT-PBS = 0,64 ± 0,03, CORT-GAS = 0,69 ± 0,07) in the hypothalamus. Both treatments did not affect dopamine and serotonin turnover in striatum, prefrontal cortex and hippocampus (see Supplementary table 2 for details).

### RNA sequencing and upstream regulator analysis

To unravel the molecular mechanisms potentially involved in GAS-mediated effects and CORT-dependent compensatory influences, we performed RNA sequencing and upstream regulator analysis. All data are reported in Supplementary Table 4 (see Supplementary Results and Supplementary Fig. 1 for a Venn diagram illustrating the unique and overlapping differentially expressed genes for all comparisons). In Table 1, for the sake of clarity, we only refer to the genes that were differentially expressed between WATER-GAS vs WATER-PBS and CORT-GAS vs WATER-GAS. In the Supplementary Information, we have provided a detailed and referenced description of our results. Below we give a more succinct overview of the most important findings and how they could be tentatively interpreted.

**Table 1:** Upstream regulator analysis - using Ingenuity - of the mRNAs that were differentially expressed in GAS-treated mice compared to controls (WATER-GAS vs WATER-PBS) (1) and in GAS-treated mice neonatally exposed to CORT compared to GAS-treated mice (CORT-GAS vs water-GAS) (2).

Upstream Regulator	WATER-GAS vs WATER-PBS (1)	CORT-GAS vs WATER-GAS (2)	Target genes
**Chemicals - endogenous mammalian**			
**beta-estradiol**	**−1**,**97**	**2**,**47**	(1): *Btg2*, *Cbl*, *Cshl1*, *Enpp2*, *Fos*, *Inpp5j*, *Sdc3*, *Sema3b*, *Srd5a1*, *Tnnt3;* (2): *Adm*, *Bcat1*, *Bmp2*, *Calb1*, *Ccng1*, *Cdkn2b*, *Chgb*, *Ctps1*, *Dlg2*, *Fgf9*, *Fmo1*, *Fnbp1*, *Fzd2*, *Gab2*, *Gsk3b*, *Hsd17b12*, *Ier2*, *Insl3*, *Lhcgr*, *Lmcd1*, *Mapt*, *Mpz*, *Myh3*, *Nedd4l*, *Nkx2–1*, *Notch3*, *Pak5*, *Pdap1*, *Pitpna*, *Ptgds*, *Pttg1*, *Pxdn*, *Pycr1*, *Pygl*, *Qsox1*, *Rapgef6*, *Slc38a2*, *Smad3*, *Snap25*, *Sstr4*, *Tgfb3*, *Thrsp*, *Timp3*, *Tpo*, *Trib1*, *Vav3*, *Zyx*
butyric acid	**—**	**2**,**16**	*Calb1*, *Ccng1*, *Col5a2*, *Gli3*, *Ly6e*, *Myh3*, *Nfatc4*, *Oas2*, *Ptgds*, *Pygl*, *Serpine1*, *Timp3*, *Uqcrq*
**dihydrotestosterone**	**−1**,**95**	**—**	*Btg2*, *Elovl7*, *Fos*, *Sdc3*, *Slc7a7*, *Srd5a1*
**dopamine**	**—**	**1**,**63**	*Adra2c*, *Bcl2l2*, *Gprc5b*, *Ncf1*, *Ppp2r2a*, *Scn1b*, *Scn9a*
**L-dopa**	**—**	**−4**,**59**	*Adra2c*, *Ajap1*, *C1qtnf12*, *C2cd2l*, *Cbr3*, *Cdc42ep2*, *Csmd3*, *Cyld*, *Dgki*, *Dlg2*, *Grid2*, *Kcne5*, *Klf16*, *Mpp6*, *Nedd4l*, *Per2*, *Plekha2*, *Ppp2r2a*, *Ptprd*, *Rell1*, *Reln*, *Slc38a2*, *Sorcs2*, *Wdr17*, *Zfhx3*
**oleic acid**	**—**	**−2**,**00**	*Ncf1*, *Npc1l1*, *Serpine1*, *Thrsp*
**Complexes**			
**IgG**	**—**	**−2**,**24**	*Adm*, *Cdkn2b*, *Csnk2b*, *Ier2*, *Serpine1*
**LH**	**—**	**2**,**00**	*Actn1*, *Btc*, *Gprc5b*, *Insl3*, *Lhcgr*, *Snap25*, *Thbs2*, *Trib1*
**IL-2**	**—**	**−1**,**76**	*Bmp2*, *Card9*, *Ccng1*, *Ccr6*, *Cmklr1*, *Csf2rb*, *Csrnp1*, *Ctps1*, *Dapk2*, *Gcnt1*, *Ly6e*, *Pus1*, *Tnfrsf4*
**Enzymes**			
CASZ1	**—**	**−1**,**96**	*Acan*, *Myh3*, *Nefl*, *Tgfb3*
EGLN1	**—**	**−1**,**98**	*Acan*, *Adm*, *Cdkn2b*, *Tgfb3*
TGM2	**—**	**1**,**91**	*Acan*, *Csrnp1*, *Dapk2*, *Ifit2*, *Ly6e*, *Mpp6*, *Oas2*
**Growth Factors**			
**FGF2**	**−1**,**93**	—	*Btg2*, *Enpp2*, *Fos*, *Prkce*, *Ptma (Includes Others)*
INHBA	**—**	**−2**,**34**	*Actn1*, *Bmp2*, *Calb1*, *Cdkn2b*, *Edar*, *Lhcgr*, *Nkx2–1*, *Pcdh9*, *Serpine1*, *Vav3*
**LEP**	**—**	**−2**,**16**	*Cps1*, *Dffa*, *Gsk3b*, *Insl3*, *Ncf1*, *Per2*, *Pln*, *Rfx1*, *Serpine1*, *Snap25*, *Thrsp*, *Timp3*
**Kinases**			
**IKBKB**	**—**	**−1**,**98**	*Bmp2*, *Cdh13*, *Timp3*, *Tnfrsf4*
**Transcription Regulators**			
KMT2A	**—**	**2**,**00**	*Gcnt4*, *Pigz*, *Trib1*, *Zmat4*
NKX2–3	**—**	**−1**,**61**	*Adm*, *Bmp2*, *Cd34*, *Cerk*, *Cryab*, *Csrnp1*, *Ly6e*, *Srpx2*, *Tmem158*
TP53	**—**	**2**,**11**	*Actn1*, *Apbb2*, *Ccng1*, *Col5a2*, *Col9a1*, *Cryab*, *Csmd3*, *Fmo1*, *Gsk3b*, *Hs3st1*, *Kcng1*, *Kifc1*, *Me2*, *Nab1*, *Ncf1*, *Otx1*, *P2rx4*, *Ptgds*, *Ptger1*, *Pttg1*, *Serpine1*, *Sirt6*, *Tgfb3*, *Thbs2*, *Timp3*, *Tmem151a*, *Zyx*
TP63	**—**	**−1**,**78**	*Adm*, *Bmp7*, *Ccng1*, *Cdkn2b*, *Kcng1*, *Notch3*, *Serpine1*, *Smad3*, *Smad4*, *Tgfb3*, *Timp3*
**Transmembrane Receptors**			
**TREM1**	**—**	**−2**,**45**	*Cdkn2b*, *Ifit2*, *Nedd4l*, *Nme7*, *Rhou*, *Tmem158*

All upstream regulators are listed with a z-score ≤ −1,50 or ≥1,50 or (see Supplementary Methods), indicating that they are inhibited or activated, respectively. The upstream regulators that could be linked to streptococcal/GAS infection, CORT exposure and/or OCD/tic disorders are indicated in bold. For each regulator, the downstream target genes are listed.

We observed that the regulatory effect of (beta-)estradiol is predicted to be inhibited in GAS mice and activated in CORT-GAS mice. Thus, while inhibition of estradiol-dependent regulation may be linked to the GAS-dependent impairments, its activation in CORT-GAS subjects may contribute to the mitigating effects of CORT. In this respect, the effects of dihydrotestosterone - a metabolite that is converted from testosterone by 5α-reductase - and fibroblast growth factor 2 (FGF2) - which has a positive effect on motor function and is upregulated by estradiol and CORT - were also predicted to be inhibited in WATER-GAS mice. Further, estradiol activates the HPA-axis, resulting in increased CORT production, reinforcing the observed beneficial effect of CORT on GAS-induced behavior. Estradiol also regulates the immune response through modulating the immunologic potency of IgG antibodies that are formed against bacteria such as GAS. In PANDAS, it is thought that IgG antibodies produced in response to GAS cross the blood brain barrier and are aimed at specific neuronal proteins in the basal ganglia, including receptors for the neurotransmitter dopamine (DA), which is converted from its precursor L-dopa. In this respect, it is interesting that our findings indicate that signaling dependent on both DA and L-dopa is dysregulated in CORT-exposed GAS mice. Furthermore, IgG-dependent regulation is predicted to be inhibited in CORT-exposed GAS mice and both estradiol and CORT increase the striatal release of DA, which further implies that increased estradiol signaling mediates the beneficial effect of CORT on GAS-induced behavior through both attenuating the IgG response and increasing DA-dependent regulation.

In addition to estradiol, signaling involving two extracellular hormones - i.e., luteinizing hormone (LH**)** and leptin (LEP) - is modulated by exposing GAS mice to CORT. LH is secreted by the pituitary gland and activates the production of sex hormones, including estradiol and testosterone. Interestingly, estradiol upregulates LH expression while its secretion is decreased by dihydrotestosterone, DA and interleukin-2 (IL-2). IL-2 is a pro-inflammatory cytokine that activates the immune response, is downregulated by beta-estradiol and increased in expression following IgG**-**mediated GAS infection. As LH-dependent regulation is predicited to be activated in CORT-exposed GAS mice while regulation downstream of IL-2 is presumably inhibited in these animals, it follows that CORT attenuates the immune response by promoting and counteracting signaling downstream of LH and IL-2, respectively. LEP, a hormone with multiple functions including appetite regulation, is predicted to be inhibited in CORT-treated GAS mice, which fits with the finding that CORT downregulates LEP expression. Further, dihydrotestosterone and beta-estradiol upregulate and downregulate LEP expression, respectively. In addition, LEP inhibits the release of DA, which fits with the finding that in CORT-treated GAS mice, the effect of LEP is inhibited while DA-dependent signaling is activated. Interestingly, LEP also plays a pro-inflammatory role through increasing IL-2 production, which suggests that the anti-inflammatory effect of CORT on GAS mice could be mediated at least in part by inhibiting LEP-dependent regulation.

Lastly, our findings point towards two proteins that represent potential targets for developing novel treatments of PANDAS, i.e., IKBKB and TREM1. IKKbeta (IKBKB) is a cytoplasmic kinase that is predicted to be inhibited in CORT-exposed GAS mice and activates NF-kappaB, a pro-inflammatory transcription factor that is an important regulator of innate immunity. Furthermore, both glucocorticoids such as CORT and estradiol negatively regulate the activity of IKBKB. In addition, IKBKB upregulates IL-2 expression. Moreover, the transmembrane protein TREM-1 is a receptor for GAS and positively mediates the GAS-induced inflammatory response, which fits with our finding that CORT exposure leads to an inhibition of TREM-1-dependent regulation in GAS mice. Given the findings for IKBKB and TREM1, we would offer that drugs inhibiting or reducing the function of both proteins could be further developed as novel PANDAS treatments (see below).

## Discussion

In accordance with our previous observations^14^, while two streptococcal injections failed to induce substantial abnormalities, four or more exposures altered behavioral, neurochemical, and immuno-histological parameters analogous to those identified in PANDAS patients. Furthermore, as predicted^3^, neonatal CORT administration compensated for most of the GAS-induced abnormalities. These effects co-occur with modifications in HPA activity, increased serum protein levels of IL-9 paralleled by IL-9+ microglia in the CNS, and changes in striatal gene expression. These results support the view that the HPA axis contributes to the regulation of immune responses ultimately modulating the severity of the abnormal phenotype. Lastly, the analysis of the differentially expressed genes in the striatum provides information regarding the potential molecular mechanisms associated with the pathogenic effects of repeated GAS exposures and the CORT-mediated compensatory role.

In line with the hypothesis that repeated GAS injections induce PANDAS-like behavioral abnormalities, GAS mice exhibited reduced sensorimotor gating and sensitivity to DOI, and increased perseverative behavior. These phenotypes constitute the preclinical analog of two of the core symptoms observed in PANDAS: impaired sensory integration^18^ and obsessive-compulsive behaviors^11^. The possibility that a failure of sensory integration relates to OCD traits in children has already been discussed elsewhere^19^. The relevance of PPI within the field of PANDAS has been substantiated by anatomical and pharmacological evidence indicating that sensorimotor gating requires an intact cortico-striato-thalamo-cortical (CSTC) circuit, generally affected in PANDAS patients^20^. Additionally, while experimental lesions of the striatal circuit impair PPI in laboratory rodents^21^, drugs acting on dopaminergic transmission modulate PPI in both rodents^22^ and humans^23^. WATER-GAS mice also exhibited increased perseverative responding in a binary-choice test, in which rodents are requested to spontaneously alternate between two choices^24^. This natural tendency requires an intact prefrontal cortex and dorsal striatum^25^. Furthermore, dopaminergic^26^ and serotonergic^27^ drugs have been shown to modulate this capability. The altered integrity of the serotonergic system in WATER-GAS mice was supported by a reduced reactivity to the pharmacological challenge with DOI and further confirmed by significant reductions in 5-HT and 5HIAA concentrations in the striatum. Moreover, in accordance with the hypothesis that anti-GAS antibodies result in central inflammatory processes^14,28^, we detected inflammatory infiltrates and ramified microglia at the level of the rostral diencephalon in GAS mice, thus supporting a role for microglia in autoimmune basal ganglia disorders like TS, OCD and PANDAS in general^29^. One aspect that warrants consideration relates to the fact that our experiments have been conducted under animal facility rearing (AFR) conditions and not under specific pathogen free (SPF) conditions, which by definition guarantee a higher level of sterility. Therefore, since immune responses may vary depending on the pathogens encountered by the organism, we cannot exclude the possibility that our results may have been different had the experiments been conducted under SPF conditions. We speculate, however, that rather than devaluing our study, this factor may confer additional validity to our results. Specifically, the higher variability of pathogens, characteristic of AFR compared to SPF, is hypothesized to introduce an additional source of uncontrolled variation in the study. The observation of remarkable effects of GAS administration, despite the presence of such unavoidable bias, further strengthens our study whereby it indirectly suggests that the long-term consequences of streptococcus administration are stronger than the background noise associated with AFR-related pathogens. Future studies are needed to clarify this aspect.

Predictably, neonatal CORT administration resulted in short- and long-term increases in basal concentrations of CORT in developing subjects^3^. In line with previous studies^30^, CORT-PBS mice exhibited remarkably reduced striatal concentrations of DA, 5HT, DOPAC and 5HIAA. Furthermore, neonatal corticosterone administration resulted, in the long-term, in reduced hypothalamic turnover of serotonin and dopamine. Alterations in brain monoamines and their turnover have already been reported in adult rodents exposed to different forms of neonatal stress^30^. Furthermore, several authors suggested that variations in brain monoamines in response to neonatal stressors may relate to deficits in prepulse inhibition^31^. Accordingly, monoamine results were functionally paralleled by behavioral alterations in PPI and sensitivity to DOI. Lastly, neonatal CORT administration resulted in increased levels of interleukin 9 (IL-9), IL-17, MCP1, MIP-1beta and in the presence of inflammatory infiltrates. IL-9 is produced by activated Th2 lymphocytes and is active on various immune cells^32^. In addition to its effect on the immune system, IL-9 has been shown to regulate cell differentiation of a hippocampal progenitor cell line^33^. Therefore, it could be hypothesized that the increased IL-9 secretion detected in CORT-PBS mice may have a role in immune regulation^34^. Analogous considerations may be proposed for IL-17, produced by Th17 lymphocytes, and for which a role in autoimmune disturbances has been identified^35^. Finally, the CORT dependent elevation in MCP1 and MIP-1beta may have also partly contributed to the compensatory effects of neonatal corticosterone administration whereby these chemokines have been shown to play a role in experimental models of autoimmunity^36^.

While both CORT and GAS exerted independent effects, the main finding of the present study resides in the modulatory role exerted by the former over the latter^9^, whereby neonatal CORT mitigated the behavioral and immunohistochemical effects of GAS. The autoimmune hypothesis of PANDAS postulates that the behavioral alterations are secondary to inflammatory phenomena at the level of the rostral diencephalon. Accordingly, besides mitigating the behavioral alterations, neonatal CORT reduced the inflammatory infiltrates and ramified microglia observed in WATER-GAS mice. Furthermore, the rare inflammatory infiltrates detected in CORT-GAS subjects contained an elevated number of IL-9+ cells. We propose that the compensatory effects of CORT on CNS inflammatory phenomena are related to the organizational long-term increase in circulating corticosteroids^9^. Such increase may act through different pathways potentially inhibiting immune activation, thereby mitigating the consequences of autoimmune-regulated phenomena. Alternatively, increased corticosteroids may regulate the production of inflammatory cytokines which could, in turn, protect the CNS. Experimental data support the latter as all GAS-treated subjects produced similar anti-GAS antibodies irrespective of glucocorticoid exposure. Therefore, we investigated whether CORT modulated the inflammatory intracerebral processes induced by repeated exposures to streptococcus. Immunological analyses performed in the serum showed that neonatal CORT increased concentrations of IL-9 in adulthood. These peripheral alterations were paralleled by IL-9+ cells with microglial morphology in the CNS. The latter may exert an anti-inflammatory role^34^ and potentially hamper the consequences of GAS administration. This finding suggests that the CORT-mediated compensatory effects would be associated with the activation of cerebral innate immunity. While current data seem to suggest that the compensatory influences of CORT occur via the activation of cytokines, the possibility that CORT modulates the production of antibodies cannot be fully excluded based on the available data. Thus, although Western Blot data indicate that the antibody response observed in CORT-GAS mice was indistinguishable from that observed in GAS mice, we note that our measure was qualitative, and not quantitative, in nature. Thus, we cannot exclude the possibility that a more detailed investigation shall identify minor between-group differences in antibody response to GAS administration. Quantitative investigations are needed to further clarify this aspect.

The upstream regulator analysis of the differentially expressed genes provides insights into the putative molecular mechanisms underlying the phenotypic abnormalities observed in GAS mice and the compensatory role of neonatal CORT. Importantly, while GAS exposures inhibited the female sex hormone (beta)-estradiol-mediated regulation of gene expression, neonatal CORT compensated for this effect. The hypothesis that estradiol exerts a regulatory role is in line with literature showing that estradiol-deficient rodents show decreased PPI^37^ and develop OCD-like behavioral abnormalities^38^. Additionally, lower estradiol levels have been shown to correlate with increased OCD symptoms in men and women^39^. Furthermore, estradiol has been reported to regulate the individual response to bacterial infections^40^ through a modulation of IgG antibodies^41^. Individuals with tics and/or OCD also have elevated serum IgG against the human dopamine D_1_ receptor (D1R)^42^. In addition, while IgG has been shown to induce PANDAS symptoms in humans, its depletion from PANDAS patient sera alleviates the symptoms^13^. Thus, the inhibitory effect exerted by CORT on IgG-regulated gene expression in GAS mice may partly contribute to its compensatory role. Lastly, in males, estradiol is produced through the conversion of the male sex hormone testosterone, which, in turn, is indirectly regulated by CORT^43^. An involvement of testosterone metabolism in regulating the consequences of GAS infection is further suggested by our finding that the effects of the testosterone metabolite dihydrotestosterone on gene expression are inhibited in WATER-GAS mice.

Furthermore, we found that CORT negatively regulates effect on gene expression mediated by Leptin (LEP), a hormone and growth factor that exerts a proinflammatory role through increasing IL-2 production by T-lymphocytes^44^. Hence, the anti-inflammatory effect of CORT could be mediated in part by an inhibition of LEP-dependent regulation. Moreover, the effect of IKKbeta (IKBKB), a kinase activating the proinflammatory transcription factor NF-kappaB (NF-kB), was predicted to be inhibited in CORT-GAS mice. IKBKB has been proposed to mediate GAS infections and the exhibition of OCD-like symptoms. Accordingly, inactivation of *Ikbkb* in mice has been shown to reduce GAS infections^45^ and normalize OCD-like behavior^46^. Lastly, CORT-GAS mice were predicted to have a reduced activation of gene expression regulated by TREM1, a receptor for GAS directly involved in inflammatory responses^47^. TREM1 has also been tested and confirmed as a drug target in the treatment of GAS-induced^48^ and other immunity-related disorders^49^. Further, it was demonstrated that CORT treatment significantly decreased the plasma expression levels of TREM-1 in febrile patients with autoimmune disease^50^. Thus, IKBKB and TREM1 represent interesting potential targets for future studies aimed at developing novel interventions to treat PANDAS, in that novel drugs could be developed that negatively regulate both targets.

**Figure 5:**
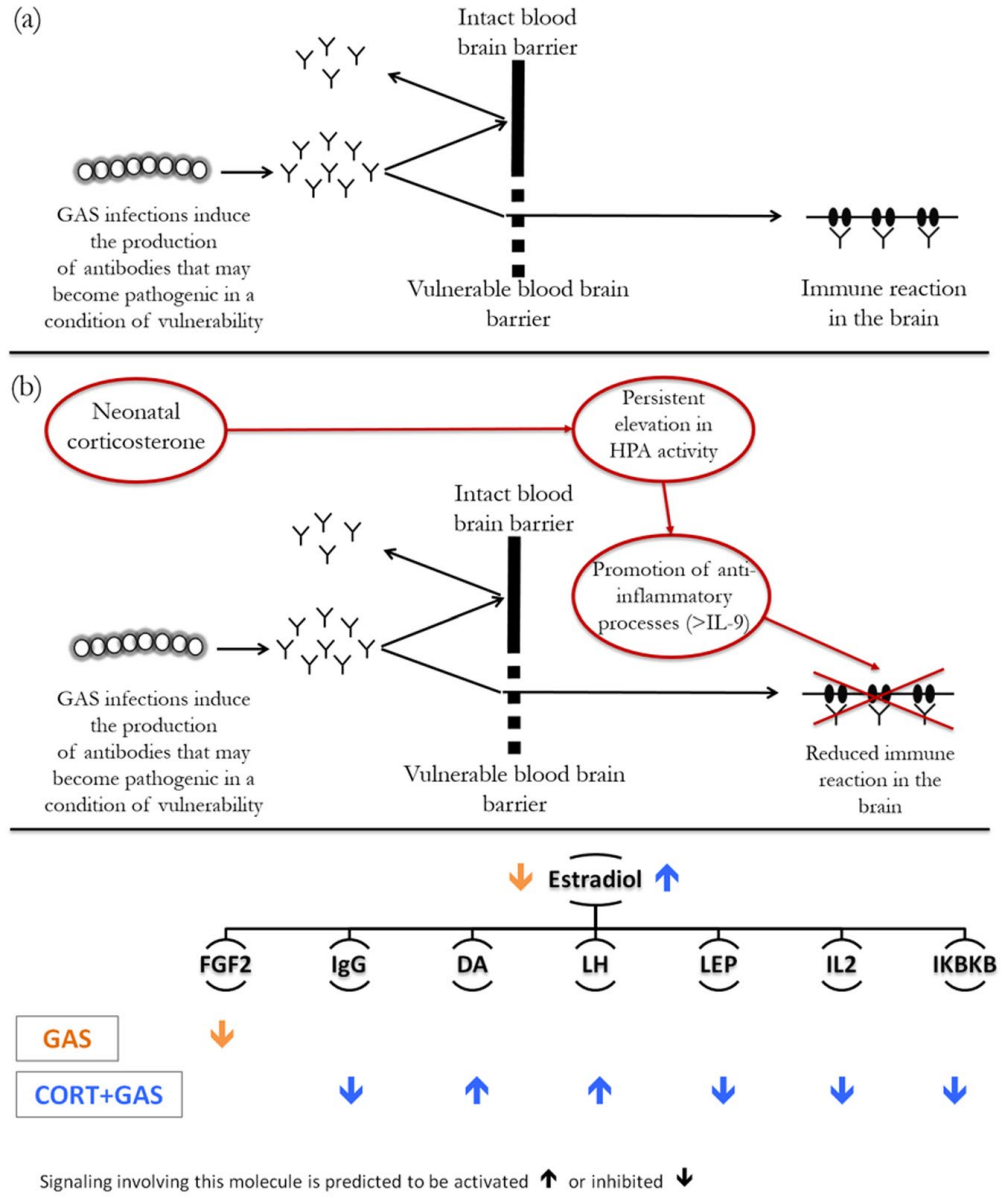
Schematic recapitulating the main topics addressed in the current study: etiological hypothesis (**a**), modulatory role of neonatal corticosterone administration (**b**), and potential molecular mechanisms involved (**c**). Specifically, in panel (a) we exemplified the etiological hypothesis linking streptococcal immunizations, antibody response and behavioral abnormalities. In panel (b) we sketched a candidate mechanism through which neonatal corticosterone may mitigate the PANDAS-like neurobehavioural abnormalities. In (**c**) we visually represented the main findings from the upstream regulator analysis. The predicted effects of GAS and CORT+ GAS are shown for signaling involving estradiol and the other signaling molecules from the analyses that are regulated by estradiol.

In conclusion, this study provides a proof of principle of the hypothesis that HPA functionality modulates the individual response to autoimmune phenomena (Fig. 5). Furthermore, we identified a potential link between neonatal experiences and the underlying molecular mechanisms that are involved in alleviating the PANDAS-related pathological phenotype.

## Methods and Materials

### Animals and rearing

All animal experiments have been performed at ISS in compliance with the European Directive 2010/63/UE and Italian Legislative Decree 26/14 on laboratory animal protection and experimentation, and authorized by the Italian Ministry of Health (Decree Nr. 217/2010-B). Thirty-six female and 18 male SJL/J young adult mice, postnatal day (PND) 56 on the day of arrival, were purchased from Charles River, Italy (Calco, Lecco, Italy). Four days after arrival, female mice were removed from their cages and housed with a male (two females per cage). After 10 days of mating, male mice were removed, dams for which mating was determined (N = 22) were housed individually in standard type-1 polycarbonate cages (33.0 × 13.0 × 14.0 cm), and checked daily at 11:00 am for delivery (see Supplementary Methods).

### Neonatal treatment

On PND0, dams were divided into two groups: control (WATER) dams (N = 11), and corticosterone-treated (CORT) dams (N = 11). CORT was supplemented in drinking water between PND1–10 as described elsewhere^30^ (see also Supplementary Methods).

### Immunization protocol

A GAS homogenate prepared as described elsewhere^12,14^ was administered through five injections interspaced by two weeks, starting on PND28 (see Supplementary Methods). To minimize litter effects, we planned not to inject siblings with the same GAS/PBS treatment. However, since litters were not perfectly balanced, out of 22 viable litters, we used littermates five times. The experimental population was composed as follows: control (WATER)-PBS, WATER-GAS, CORT-PBS, and CORT-GAS.

### Experimental design

The experimental design (See Fig. 1) entailed repeated behavioral testing, blood sampling for the evaluation of the immune response to the injection protocol, blood sampling for CORT level determination in basal conditions, and brain sampling/sectioning for neurochemical and immunohistochemical assays, and RNA sequencing. We conducted the experiments in two batches:

- batch 1: WATER-PBS, N = 8; WATER-GAS, N = 8; CORT-PBS, N = 10; CORT-GAS, N = 12.
- batch 2: WATER-PBS, N = 5; WATER-GAS, N = 6 mice; CORT-PBS, N = 7; and CORT-GAS, N = 7.

### Physiological indicators of the efficacy of experimental treatments

To determine the efficacy of CORT and GAS treatments, we quantified the short- and long-term effects of CORT administration on corticosterone concentrations and of repeated exposures to GAS on the formation of antibodies. We thus evaluated: CORT concentrations in hair on PND23, and in plasma in adulthood; and anti-GAS antibodies in serum samples following the fourth GAS injection (Supplementary Methods for details).

### Behavioral testing

The first test battery, performed one week after the second injection (week 7), entailed: *elevated zero-maze* (day 1) to evaluate potential effects on anxiety-like behavior; *pre-pulse inhibition* (*PPI*, day 2 for habituation and day 3 for testing) to demonstrate that GAS affected sensorimotor integration processes characteristic of PANDAS and OCD; and the evaluation of spontaneous locomotion through an automated scoring system (day 4). While elevated zero-maze and locomotion were assessed in the animal facility, PPI was conducted in a separate testing room.

The same animals were screened one week after the fourth injection (week 11), in a behavioral test battery entailing five tests, performed in the following sequence: *elevated plus maze*, *rotarod* (to control for potential effects of treatments on motor coordination), and *PPI habituation* on day 1; *PPI testing* on day 2; *t-maze*, to evaluate repetitive behaviors, (days 3–7); *locomotor activity* (days 7–8); individual response (exhibition of *head twitch*) to the serotonergic agonist DOI to evaluate the functionality of the serotonergic system. The behavioral test procedures are detailed in the Supplementary Methods.

### Immune activation in response to experimental treatments

We evaluated inflammatory processes in the central nervous system (CNS) through immunohistochemistry and peripheral cytokines in plasma samples. For immunohistochemistry, coronal cryosections (10 μm thickness) were cut from whole brains from 5 CORT-PBS and 5 CORT-GAS treated mice and stored at −80 °C. As previously described^14^, hematoxylin and eosin staining was performed on all the examined brain samples (1 cryosection every 15 cut cryosections) in order to assess the presence of potential inflammatory infiltrates and any evident tissue alteration. Air-dried cryosections were used for immunohistochemistry assessment of the level of microglia activation (Iba1) and for the immunolocalization of TNF and IL-9 cell expression (see Supplementary Methods). Cytokine concentrations in the serum were determined through the Bio-Plex Cytokine Assay (23-Plex, Bio-Rad) following the producer’s protocol (see Supplementary Methods for details).

### Monoamine measurements

To confirm previous evidence that GAS administration alter brain monoamines, and further confirm the similarity between our model and PANDAS, we evaluated 5-HT, DA and their metabolites, i.e. 5-hydroxyindole acetic acid (5-HIAA), and 3,4-dihydroxyphenylacetic acid (DOPAC) in prefrontal cortex, striatum, hippocampus, hypothalamus and cerebellum. These were quantified by a modified method of HPLC combined with an electrochemical detector as previously described^14^. See Supplementary Methods for further details.

### RNA sequencing

Using RNA sequencing, we quantified total RNA levels in 22 mice (5 WATER-PBS, 5 WATER-GAS, 6 CORT-PBS and 6 CORT-GAS). Further details are provided in the Supplementary Methods. Briefly, the final sets of differentially expressed genes comprised those that were identified as such – fold change ≥1.2 and P-value < 0.01 – by at least two of the four used methods. The Venn diagram of the final sets of differentially expressed genes for all comparisons was built with Venny (see Supplementary Methods). For genes that showed differential expression, upstream regulator analysis was performed using the Ingenuity Pathway Analysis software (IPA), version 00.06 (Ingenuity Systems Inc., Redwood City, CA). Further details are provided in the Supplementary Methods.

### Statistical analyses

Statistical analyses were conducted using the software Statview 5.0 (Abacus Concepts, USA). The experimental design entailed two between-subjects factors (neonatal treatment: WATER vs. CORT; and PBS/GAS treatment, two levels: PBS vs. GAS) and one within-subject factor (repeated measures with a variable number of levels, depending on the specific parameter). Thus, the experimental model consisted of a 2 (neonatal treatment) × 2 (PBS/GAS treatment) × k (repeated measurements) ANOVA for split-plot designs. Tukey’s post hoc tests were used for between-group comparisons. Statistical significance was set at *p* < 0,05. Finally, to evaluate whether experimental subjects met the criterion for T-maze paradigm, the observed phenotype has been compared to the threshold through one-sample T- tests.

## Electronic supplementary material

Supplementary methods and results

## References

[1] Bateson P (2004). Developmental plasticity and human health. Nature.

[2] Heim C, Plotsky PM, Nemeroff CB (2004). Importance of studying the contributions of early adverse experience to neurobiological findings in depression. Neuropsychopharmacology.

[3] Macrì S, Zoratto F, Laviola G (2011). Early-stress regulates resilience, vulnerability and experimental validity in laboratory rodents through mother-offspring hormonal transfer. Neurosci Biobehav Rev.

[4] Macrì S, Wurbel H (2006). Developmental plasticity of HPA and fear responses in rats: a critical review of the maternal mediation hypothesis. Horm Behav.

[5] Plamondon A (2015). Spatial working memory and attention skills are predicted by maternal stress during pregnancy. Early Hum Dev.

[6] Bakker JM (2000). Neonatal dexamethasone treatment increases susceptibility to experimental autoimmune disease in adult rats. J Immunol.

[7] Meagher MW (2010). Neonatal maternal separation alters immune, endocrine, and behavioral responses to acute Theiler’s virus infection in adult mice. Behav Genet.

[8] Wick G, Hu Y, Schwarz S, Kroemer G (1993). Immunoendocrine communication via the hypothalamo-pituitary-adrenal axis in autoimmune diseases. Endocr Rev.

[9] Levine S, Strebel R, Wenk EJ, Harman PJ (1962). Suppression of experimental allergic encephalomyelitis by stress. Proc Soc Exp Biol Med.

[10] Levine S, Wenk EJ, Muldoon TN, Cohen SG (1962). Enhancement of experimental allergic encephalomyelitis by adrenalectomy. Proc Soc Exp Biol Med.

[11] Swedo SE (1998). Pediatric autoimmune neuropsychiatric disorders associated with streptococcal infections: clinical description of the first 50 cases. Am J Psychiatry.

[12] Hoffman KL, Hornig M, Yaddanapudi K, Jabado O, Lipkin WI (2004). A murine model for neuropsychiatric disorders associated with group A beta-hemolytic streptococcal infection. J Neurosci.

[13] Yaddanapudi K (2010). Passive transfer of streptococcus-induced antibodies reproduces behavioral disturbances in a mouse model of pediatric autoimmune neuropsychiatric disorders associated with streptococcal infection. Mol Psychiatry.

[14] Macrì S (2015). Mice repeatedly exposed to Group-A beta-Haemolytic Streptococcus show perseverative behaviors, impaired sensorimotor gating, and immune activation in rostral diencephalon. Sci Rep.

[15] Martino D, Dale RC, Gilbert DL, Giovannoni G, Leckman JF (2009). Immunopathogenic mechanisms in tourette syndrome: A critical review. Mov Disord.

[16] Orlovska, S. *et al*. Association of Streptococcal Throat Infection With Mental Disorders: Testing Key Aspects of the PANDAS Hypothesis in a Nationwide Study. *JAMA Psychiatry*, 10.1001/jamapsychiatry.2017.0995 (2017). 10.1001/jamapsychiatry.2017.0995 PMC5710247 28538981

[17] Dale RC, Brilot F (2012). Autoimmune basal ganglia disorders. J Child Neurol.

[18] Swerdlow NR (2013). Update: studies of prepulse inhibition of startle, with particular relevance to the pathophysiology or treatment of Tourette Syndrome. Neurosci Biobehav Rev.

[19] Stein DJ (2000). Advances in the neurobiology of obsessive-compulsive disorder. Implications for conceptualizing putative obsessive-compulsive and spectrum disorders. Psychiatr Clin North Am.

[20] Ganos C, Roessner V, Munchau A (2013). The functional anatomy of Gilles de la Tourette syndrome. Neurosci Biobehav Rev.

[21] Baldan Ramsey LC, Xu M, Wood N, Pittenger C (2011). Lesions of the dorsomedial striatum disrupt prepulse inhibition. Neuroscience.

[22] Mansbach RS, Geyer MA, Braff DL (1988). Dopaminergic stimulation disrupts sensorimotor gating in the rat. Psychopharmacology (Berl).

[23] Csomor PA (2008). Haloperidol differentially modulates prepulse inhibition and p50 suppression in healthy humans stratified for low and high gating levels. Neuropsychopharmacology.

[24] Deacon RM, Rawlins JN (2006). T-maze alternation in the rodent. Nat Protoc.

[25] Lalonde R (2002). The neurobiological basis of spontaneous alternation. Neurosci Biobehav Rev.

[26] Irwin J, Tombaugh TN, Zacharko RM, Anisman H (1983). Alteration of exploration and the response to food associated cues after treatment with pimozide. Pharmacol Biochem Behav.

[27] Jaffard R (1991). Effects of tianeptine on spontaneous alternation, simple and concurrent spatial discrimination learning and on alcohol-induced alternation deficits in mice. Behav Pharmacol.

[28] Spinello C, Laviola G, Macrì S (2016). Pediatric Autoimmune Disorders Associated with Streptococcal Infections and Tourette’s Syndrome in Preclinical Studies. Front Neurosci.

[29] Frick L, Pittenger C (2016). Microglial Dysregulation in OCD, Tourette Syndrome, and PANDAS. J Immunol Res.

[30] Zoratto F, Fiore M, Ali SF, Laviola G, Macrì S (2013). Neonatal tryptophan depletion and corticosterone supplementation modify emotional responses in adult male mice. Psychoneuroendocrinology.

[31] Niwa M, Matsumoto Y, Mouri A, Ozaki N, Nabeshima T (2011). Vulnerability in early life to changes in the rearing environment plays a crucial role in the aetiopathology of psychiatric disorders. Int J Neuropsychopharmacol.

[32] Demoulin JB, Renauld JC (1998). Interleukin 9 and its receptor: an overview of structure and function. Int Rev Immunol.

[33] Mehler MF, Rozental R, Dougherty M, Spray DC, Kessler JA (1993). Cytokine regulation of neuronal differentiation of hippocampal progenitor cells. Nature.

[34] Elyaman W (2009). IL-9 induces differentiation of TH17 cells and enhances function of FoxP3+ natural regulatory T cells. Proc Natl Acad Sci USA.

[35] Tabarkiewicz J, Pogoda K, Karczmarczyk A, Pozarowski P, Giannopoulos K (2015). The Role of IL-17 and Th17 Lymphocytes in Autoimmune Diseases. Arch Immunol Ther Exp (Warsz).

[36] Youssef S, Wildbaum G, Karin N (1999). Prevention of experimental autoimmune encephalomyelitis by MIP-1alpha and MCP-1 naked DNA vaccines. J Autoimmun.

[37] van den Buuse M, Simpson ER, Jones ME (2003). Prepulse inhibition of acoustic startle in aromatase knock-out mice: effects of age and gender. Genes Brain Behav.

[38] Hill RA (2007). Estrogen deficient male mice develop compulsive behavior. Biol Psychiatry.

[39] Boon WC, Horne MK (2011). Aromatase and its inhibition in behaviour, obsessive compulsive disorder and parkinsonism. Steroids.

[40] Garcia-Gomez E, Gonzalez-Pedrajo B, Camacho-Arroyo I (2013). Role of sex steroid hormones in bacterial-host interactions. Biomed Res Int.

[41] Ercan A (2017). Estrogens regulate glycosylation of IgG in women and men. JCI Insight.

[42] Cox CJ (2015). Antineuronal antibodies in a heterogeneous group of youth and young adults with tics and obsessive-compulsive disorder. J Child Adolesc Psychopharmacol.

[43] Lescoat G, Lescoat D, Garnier DH (1982). Influence of adrenalectomy on maturation of gonadotrophin function in the male rat. J Endocrinol.

[44] Lord GM (1998). Leptin modulates the T-cell immune response and reverses starvation-induced immunosuppression. Nature.

[45] Hsu LC (2011). IL-1beta-driven neutrophilia preserves antibacterial defense in the absence of the kinase IKKbeta. Nat Immunol.

[46] Krabbe G (2017). Microglial NFkappaB-TNFalpha hyperactivation induces obsessive-compulsive behavior in mouse models of progranulin-deficient frontotemporal dementia. Proc Natl Acad Sci USA.

[47] Tsatsaronis JA, Walker MJ, Sanderson-Smith ML (2014). Host responses to group a streptococcus: cell death and inflammation. PLoS Pathog.

[48] Horst SA (2013). Prognostic value and therapeutic potential of TREM-1 in Streptococcus pyogenes- induced sepsis. J Innate Immun.

[49] Murakami Y (2009). Intervention of an inflammation amplifier, triggering receptor expressed on myeloid cells 1, for treatment of autoimmune arthritis. Arthritis Rheum.

[50] Lin CH (2016). Prospective Evaluation of Procalcitonin, Soluble Triggering Receptor Expressed on Myeloid Cells-1 and C-Reactive Protein in Febrile Patients with Autoimmune Diseases. PLoS One.

