# Supplementary material for: Neonatal corticosterone mitigates autoimmune neuropsychiatric disorders associated with streptococcus in mice
Source: Sci Rep. 2018 Jul 5;8:10188. doi: 10.1038/s41598-018-28372-3 (PMC6033871; doi:10.1038/s41598-018-28372-3)
Supplement: Supplementary file 1 — Supplementary methods and results [file 41598_2018_28372_MOESM1_ESM.pdf]

Neonatal corticosterone mitigates autoimmune neuropsychiatric disorders associated with streptococcus in mice

Authors: Simone Macrì[1,€,*], Chiara Spinello[1,€], Joanna Widomska[2,€], Roberta Magliozzi[5,6,€], Geert Poelmans[3,4], Roberto William Invernizzi[7], Roberta Creti[8], Veit Roessner[9], Erika Bartolini[10], Immaculada Margarit[10], Jeffrey Glennon[4], Giovanni Laviola[1]

[*]Corresponding Author:
Centre for Behavioural Sciences and Mental Health, Istituto Superiore di Sanità, Viale Regina Elena, 299, I-00161 Roma, Italy
Tel: +39-06-4990-3285
Email: simone.macri@iss.it

[€] Equally contributed to the manuscript

[1]Centre for Behavioural Sciences and Mental Health, Istituto Superiore di Sanità, Roma, Italy; [2]Dept. Cognitive Neuroscience, Radboud University Medical Center, Nijmegen, The Netherlands; [3]Dept. Human Genetics, Radboud University Medical Center, Nijmegen, The Netherlands; [4]Dept. Molecular Animal Physiology, Donders Institute for Brain, Cognition and Behaviour, Radboud Institute for Molecular Life Sciences (RIMLS), Radboud University, Nijmegen, The Netherlands; [5]Neurology B, Dept. of Neurological, Biomedical and Movement Sciences, University of Verona, Italy; [6]Division of Brain Sciences, Faculty of Medicine, Imperial College London, UK; [7]IRCCS-Istituto di Ricerche Farmacologiche "Mario Negri", Milano, Italy; [8]Respiratory and Systemic Bacterial Diseases Section, Dept. of Infectious, Parasitic, and Immune-mediated Diseases, Istituto Superiore di Sanità, Roma, Italy; [9]Dept. of Child and Adolescent Psychiatry, Faculty of Medicine of the TU Dresden, Germany; [10]GSK, Siena, Italy

Short title – Neonatal stress favors resilience towards autoimmunity

Keywords – PANDAS; Immunity; Animal Models; Perseverative Behavior; Pre-Pulse Inhibition; RNA sequencing

Supplementary Methods

Animals and rearing

Upon arrival, males were housed individually in type-1 polycarbonate cages (33.0×13.0×14.0 cm), while females were housed in groups of 6 in type-3 polycarbonate cages (42.0×26.0×15.0 cm). All cages were equipped with sawdust bedding, an enrichment bag (Mucedola, Settimo Milanese, Italy), metal top and *ad libitum* water and food pellets (Mucedola, Settimo Milanese, Italy). Mice were maintained on a reversed 12-h-light-dark cycle (light on at 7:00 PM) in an air-conditioned room (temperature 21 ± 1°C and relative humidity 60 ± 10%).

Neonatal treatment

Starting on PND1, were supplemented with corticosterone (80 μg/ml) in their drinking water. CORT administration was maintained until PND10. CORT concentration was selected based on previous literature (Macrì *et al*, 2007; Zoratto *et al*, 2011; Zoratto *et al*, 2013). CORT-exposed mice drank on average 13,9±0,49 ml of CORT solution; this resulted in an average daily intake of 1,11±0,039 mg. Except for the solution in the drinking bottle, neonatal environmental conditions were identical among groups. Subjects were left undisturbed until PND9, when pups were weighed and sexed. In addition, from PND9 onwards, cages were regularly cleaned once a week. On PND23, individuals were weaned and housed in same-sex, same-treatment groups of two or three individuals. On PND27, all mice were marked through ear clipping.

Immunization protocol

After preparation, GAS homogenate was stored at -70°. A blood agar plate was used to inoculate a sample of homogenate (2.5 μl), to verify that it contained no viable bacteria. The immunization protocol, described by Hoffman et al (Hoffman *et al*, 2004) and adopted in our previous study (Macrì *et al*, 2015), comprised five injections interspaced by a time interval of two weeks, starting on PND28. During the first injection, PBS mice were injected subcutaneously (s.c.) with 125 μL of an emulsion

(1:1), containing PBS and Complete Freund's adjuvant (CFA; Sigma-Aldrich, Milano, Italy). GAS mice were injected with 125 μL of the same emulsion (CFA:PBS), containing 5 μL of GAS homogenate (0.52 mg/ml of total protein as determined by Bradford Assay, Bio-Rad). All mice were then treated four additional times at two-weeks intervals with 125 μL of vehicle – an emulsion containing PBS and Incomplete Freud's Adjuvant (IFA; Sigma-Aldrich, Milano, Italy) – for PBS mice, or 125 μL of vehicle and 5 μL of GAS homogenate for GAS mice. To prepare the PBS/adjuvant emulsions, we used the vortex method described by Flies and Chen (Flies and Chen, 2003).

Experimental Design

Experimental tests were distributed among batches as follows: Batch 1 was used for behavioral testing (elevated 0-maze, locomotor activity, elevated plus maze, rotarod, T-maze, pre-pulse inhibition, and observation of DOI-induced behavioral responses), basal CORT concentrations, antibody response, plasma cytokines and immunohistochemistry at sacrifice (fourth injection); Batch 2 was used to evaluate hair CORT concentrations at weaning, brain monoamines at sacrifice, and RNA sequencing. All animals were sacrificed two weeks after the fifth injection.

Physiological indicators of the efficacy of experimental treatments

<u>Hair and blood sampling</u>

To evaluate the effects of neonatal and adult treatment on HPA activity, we evaluated CORT levels both at weaning (through non-invasive CORT analysis of hair samples) and at the end of the study through trunk blood collection. Blood samples were also used for the determination of antibodies. Hair samples were collected through shaving a small portion of hair on the back of the subjects. Trunk blood serum samples (~200 μL) were collected after the fourth boost of immunization, at the moment of the sacrifice. Blood samples were allowed to clot at room temperature for 4 hours, centrifuged at 3000 rpm for 15 minutes. The serum was transferred into Eppendorf tubes and maintained at -80°C until the biochemical assays were performed. CORT concentration was assessed using a commercial radioimmunoassay (RIA) kit (ICN Biomedicals, Costa Mesa, CA). Vials were counted for 2 minutes in a

gamma counter (Packard Minaxi Gamma counter, Series 5000). The procedures for washing and steroid extraction followed the protocol described by Gao and colleagues (Gao *et al*, 2013), who analyzed hair steroids with liquid chromatography tandem mass spectrometry (LC-MS/MS). One change was made to the protocol: the dry residue was resuspended using 175μL distilled water. Afterward, 100μL of the medium was injected into a Shimadzu HPLC system (Shimadzu, Canby, OR, USA) coupled to an AB Sciex API 5000 Turboion-spray1triple quadrupole tandem mass spectrometer equipped with Atmospheric Pressure Chemical Ionization (APCI) Source (AB Sciex, Foster City, CA, USA). The system was controlled by AB Sciex Analyst1 software (version 1.5.1). The lower limit of detection was ~0.1pg/mg. Intra- and inter-plate coefficients of variance ranged between 3.7–8.8%. All samples were prepared and analyzed within the same time period in order to prevent batch effects.

Analysis of anti-Group A Streptococcal antibodies in serum samples

GAS homogenates obtained as described above were size-separated by SDS-PAGE (4–12% acrylamide) under reducing conditions and electroblotted onto nitrocellulose membranes. Immunostaining was performed by blocking the membrane overnight with 3% (w/v) skimmed milk in TPBS (0.1% Tween in PBS) and incubating for 2 h with sera from mice of all experimental groups (all sera were diluted 1: 200). After 3 washes with TPBS, the membrane was incubated with HRP-conjugated secondary antibody (1:1000), washed again with TPBS and PBS, and developed with a chromogenic substrate.

Behavioral testing

Elevated 0-maze

Mice were tested for anxiety-related behaviors on the elevated 0-maze, which imposes an approach-avoidance conflict. The apparatus consisted of a 5.5 cm wide circular runway made of black plastic with an outer diameter of 46 cm and placed 40 cm above the floor. Two closed sectors, positioned opposite to each other, were protected by 16 cm high walls made of transparent Plexiglas; the two remaining sectors were unprotected (open sectors). Mice were tested between 10:00 and 12:00 am directly in the

housing room, under dim light. At the beginning of the experimental session, the animal was gently positioned into one of the two closed sectors and allowed to explore the apparatus for 5 minutes. Between experimental sessions, the apparatus was cleaned with an alcohol/water solution (50%). Data acquired through a camera (Sony, Handycam, DCR-SX21E) were saved on a personal computer (Dell, Dell Precision T1600) and scored offline using the "Observer 10.5" (Noldus, Wageningen, The Netherlands). For each animal, we detected spatiotemporal variables (time spent and the number of entries in each sector), and behavioral variables in frequency and duration (*rearing*, *grooming*, *stretched attend posture (SAP)*, and *head dipping*). The total number of entries was considered as an index of general activity, while time spent in the closed sectors was used as a measure of anxiety (Zoratto *et al*, 2011).

Pre-pulse inhibition (PPI)

Apparatus: The apparatus (Med Associates inc. St Albans, VT, United States of America (Olivier *et al*, 2001)) consisted of an acoustic stimulator (ANL-925, Med Associates inc.) and a platform with a transducer amplifier (PHM-250-60, Med Associates inc.), and was positioned in a foam-lined isolation chamber (ENV-018S, Med Associates inc.), defined as startle chamber. The presence of a red light and a fan, both enclosed in the chamber, guaranteed dimmed lighting and ventilation. The platform was enclosed in a perforated compartment to ensure that the experimental subjects remained on it. Experimental data were acquired through dedicated software (SOF-815, Med Associates inc.).

Procedure: During habituation, each mouse was positioned individually inside the startle chamber, and left undisturbed (without any stimulus) for five minutes. On the following day, mice were placed again inside the startle chamber for testing. At the beginning of the experimental session, mice were exposed to a white noise (62dB) for five minutes. After acclimation, mice were then exposed to a sequence of ten trials (pulses of 120dB), that were interspaced by an average inter-trial interval of 15 s (block I). Then, an 8-min long session started (block II). This session entailed 28 trials comprising four different types of trials that were presented in a pseudorandomized order. Trials were defined as follows:

prepulse alone (one trial for prepulse intensity), prepulse plus pulse (four trials for prepulse intensity), startle alone (four trials) and no stimulation (four trials). The inter-trial interval varied between 10-s and 20-s to avoid habituation. Each trial started with a null period of 50 ms, followed by a prepulse noise. The intensity of the prepulse varied among four different values, represented by 67, 70, 73 or 76 dB. Following the prepulse, a startle stimulus was presented. The startle stimulus, as described previously, was constituted by a white noise of 40ms long and with an intensity of 120 dB. Prepulse and pulse stimuli were interspaced by an inter-stimulus period of 100 ms. The galvanic response was considered as the dependent variable and was measured 65 ms following the onset of the startle. Prepulse inhibition (PPI) was measured as PPI= [(A-B)/A*100], wherein A is the Galvanic reflex registered after the startle stimulus alone, and B is the reflex registered in response to the startle in prepulse plus pulse trials.

Locomotor activity

After the second and the fourth injection, mice were monitored for spontaneous locomotion over a 24-hr period. General locomotion was evaluated in experimental cages identical to the home cages and positioned on a rack within the housing room. Six hours before the beginning of the registration session, mice were housed individually. Locomotor activity was then monitored for 24 consecutive hours through an automated device using small passive infrared sensors positioned on the top of each cage (ACTIVISCOPE system, NewBehavior Inc., Zurich, Switzerland) (Dell'Omo *et al*, 2002; Proietti Onori *et al*, 2014). The movement of the mice was detected with sensors operating at a frequency of 20 events per second (20 Hz). When mice were sleeping or inactive, the sensor did not detect any movement. Average scores measured in 60-min intervals and expressed as count per minute (cpm) were detected for each mouse using dedicated software (NewBehavior Inc., Zurich, Switzerland). At the end of the test, mice were relocated to their original home cages.

Accelerating rotarod test

Accelerating rotarod test was performed to evaluate mouse balance and motor coordination, by measuring the latency to fall from a rotating cylinder (Basile, Comerio, Italy). The apparatus consisted of a rotating rod (3 cm in diameter), on which mouse had to walk to avoid falling. To prevent harm to subjects, the cylinder was positioned 13 cm above the falling surface. A timer connected to a switch was used to record the latency, and the timer stopped when the mouse fell from the rod. During each trial, the rate of rotation progressively increased from 4 to 40 revolutions per minute (r.p.m.) over 4 minutes. Mice performed three consecutive trials interspaced by a time interval of 10 minutes. The trial stopped when the mouse fell down from the cylinder, or when it reached the cut-off (4 minutes). The test was performed in an experimental room, adjacent to the housing room, between 10:00 and 14:00, where mice were carried to one hour before the beginning of the experimental session.

T-maze

Animals were screened for perseverative behaviors in the T-maze test. The experimental apparatus consisted of an enclosed T-shaped maze, composed of three equally sized arms (50x16 cm). Ten sessions were performed during five consecutive days (2 sessions per day), always in the housing room. The experimental session, consisting of two choice trials, started with the mouse positioned in the starting compartment, facing the wall of the apparatus. Then, the subject was allowed to explore the apparatus for two minutes. As soon as the animal completed the trial (entering one of the two alternative arms), such instance was scored as the first choice and the door of the arm was closed. After a few seconds, the animal was gently removed from the arm, placed again in the starting compartment, and allowed to perform a second choice trial. If the subject entered the arm opposite to the previously chosen one, an instance of alternation was scored. Alternatively, if the mouse re-entered the same arm, an instance of perseveration was scored. The percentage of alternations, measured as the number of alternations divided by the number of completed sessions times 100, was scored for each mouse. In a

T-maze, rodents have the natural tendency to alternate their choice of the arm (spontaneous alternation) (Deacon and Rawlins, 2006).

Elevated plus maze

The elevated plus maze test was performed after the fourth injection, to evaluate the exploration of an environment imposing on the animal an approach-avoidance conflict. The apparatus, elevated to a height of 40 cm above the floor level, comprised two open arms (27x5x0.25 cm) and two closed arms (27x5x15 cm), extended from a common central platform (5x5 cm). The floor and walls of the apparatus were both made of Plexiglas (black floor, transparent walls). The test was performed in an experimental room between 10:00 and 14:00, where mice were carried to one hour before the beginning of the experimental session. At the beginning of the test, mice were positioned gently in the central platform of the apparatus facing one of the two closed arms, and allowed to explore the maze for 5 minutes. The test was performed under dim illumination, and the apparatus was cleaned with an alcohol solution (50%) after each animal was tested. All sessions were recorded by a video camera (Sony, Handycam, DCR-SX21E), saved on a personal computer (Dell, Dell Precision T1600), and scored offline using the "Observer 10.5" (Noldus). For each animal, we detected spatiotemporal variables (time spent and number of entries in each sector), and behavioral variables in frequency and duration. In particular, we scored: *rearing* (head raising standing on hind legs), *grooming* (self-cleaning with mouth or paws), *stretched attend posture (SAP)* (stretched posture with the head and two or three paws on the open arm and retraction to previous position) and *head dipping* (downward movement of the head towards the floor while on the open arms).

Individual reactivity to serotonergic agonist (DOI)

In order to observe the individual reaction to a serotonergic agonist, we evaluated the behavioral response to the administration of the 5-HT2a agonist DOI (2,5-dimethoxy-4-iodoamphetamine, Sigma-Aldrich, St. Louis, MO, USA, 5 mg/kg). DOI was dissolved in saline (NaCl 0,9%) and administered i.p.

at a volume of 1 ml/100 g body weight (Egashira *et al*, 2011), to the animals of batch 1 after the fifth injection (week 13). Starting 5 minutes after the injection, animals were video recorded for the following 10 minutes, using a video camera mounted on the back of the home cages. To prevent the occlusion of the visual space targeted by the video camera, the day before the test all animals were housed individually in cages where only a thin layer of bedding was left. The video-recording apparatus was composed of 32 video cameras (Simon TLC, PRS Italia, Rome, Italy) positioned on the back of the rack and connected to a DVR system (Gifran, 16 Channels Realtime, H.264 Full D1). Each black and white video camera (resolution: 420 lines, illumination: 0.01 Lux, lens: 3.6 mm) was positioned on a panel at a distance of 4.0 cm from the short side of the cages, with the centre of the lens focused on the cage to allow the tracking of animal movements and behaviors (see (Proietti Onori *et al*, 2014) for details). Behavioral responses were then scored using a computer with a dedicated software (The Observer 10.5, Noldus). Frequency and duration of two behavioral responses were scored: *head twitch*, a rapid and violent head shaking (Canal and Morgan, 2012), and *skin jerk*, a muscle contraction of the body longer in duration than the twitch (Proietti Onori *et al*, 2014).

Immunohistochemistry

For immunohistochemistry, air dried cryosections were passed in 70%, 95%, and 100% ethanol, and after rehydration with PBS and 20-min incubation with 0.3% H_2O_2 in PBS to eliminate endogenous peroxidase activity, sections were pre-incubated with 10% of normal sera and incubated at 4°C overnight with rabbit polyclonal IBA1 antibody (Wako-Chem, Japan), or goat polyclonal TNF antibody (R&D System, Minneapolis, Minn) or IL-9 (diluted in PBS containing 5% normal donkey serum, followed by incubation with biotinylated secondary antibodies (Jackson ImmunoResearch Laboratories, Suffolk, UK) visualized with the avidin-biotin horseradish peroxidase complex (ABC Vectastain Elite kit, Vector Laboratories, Burlingame, CA, USA) and 3,3 diaminobenzidine (Sigma Chemical, St. Louis, MO, USA). All sections were counterstained with haematoxylin, sealed with Canada balsam and viewed and photographed with an Axiophot Zeiss microscope (Germany) equipped with an Axiocam digital camera using the Axiovision 4AC software. Qualitative analysis of the presence and distribution of

inflammatory cells was performed by a trained observer who did not perform the cutting (R.M.) and was blind to the treatment across all the different brain areas.

Cytokine concentrations in the serum were determined through the Bio-Plex Cytokine Assay (23-Plex, Bio-Rad). Briefly, differentially stained microbeads, each type of which is coated with antibodies recognizing different cytokines, were incubated with mouse sera, washed, and incubated with biotinylated detection antibodies. Then the beads with the sandwich between antibodies and cytokines were washed, incubated with streptavidin-PE, washed again, and read in the Bio-Rad array. We determined cytokine concentration as an average of two replicates per serum.

Monoamine measurements

In order to collect brain samples, mice were rapidly decapitated. Samples collected for evaluation of brain monoamines by HPLC analyses were immediately sectioned on ice to obtain five sections: prefrontal cortex, striatum, hippocampus, hypothalamus, and cerebellum. All samples were flash frozen and then stored at -80°C until analysis. Brain samples collected for immunohistochemical analyses and RNA sequencing were collected after decapitation, kept intact, flash frozen and stored at -80°.

Briefly, each brain region was weighed and a measured volume (10% W/V) of 0.1 N perchloric acid containing 0.05% Na2S2O5 and 0.1% Na2EDTA was added. The tissue was then disrupted by ultrasonication, centrifuged (10,000 × g; 5 minutes), and 100 µl of the supernatant was removed and filtered through 0.45 μm PVDF syringe filters (Perkin-Elmer, Italy). Aliquots of 20 µl were injected directly onto the HPLC/EC system by a refrigerated (5°C) autosampler (MIDAS, Spark-Holland, The Netherlands) for the separation of 5-HT, DA, 5-HIAA, and DOPAC through a Supelcosil LC-18DB, 3 μm (75 × 3.0 mm) analytical column (Supelchem, Italy), thermostated at 40°C. The mobile phase consisted of sodium acetate anhydrous (5 g/L), citric acid (4.5 g/L), sodium octane sulphonate (100 mg/L), Na2EDTA dihydrate (112 mg/L); CH3OH 7%. Monoamines and metabolites were measured by a Coulochem II electrochemical detector (Dionex, Switzerland) equipped with a 5011 electrochemical cell (E1 and E2 potentials were 0 and +350 mV, respectively). A data system (Azur 4.6,

Datalys, France) was used to calculate the concentration of analytes based on calibration curves prepared daily with appropriate concentrations of pure standards.

RNA sequencing

Total RNA was extracted from striatum tissue using the Qiagen AllPrep Kit (Qiagen, Hamburg, Germany), according to the manufacturer's instructions. RNA was further quantified using the NanoDrop 1000 Spectrophotometer and Qubit Fluorometer (Thermo Fisher Scientific, MA, USA) and RNA integrity was assessed with the 2200 TapeStation Instrument (Agilent Technologies, CA, USA). A total of 500 ng RNA was used for library preparation with the TruSeq Stranded Total RNA with Ribo-Zero Gold sample preparation kit (Illumina, CA, USA). Successfully prepared cDNA libraries were then submitted for sequencing at the Department of Human Genetics, Radboud University Medical Center, Nijmegen, The Netherlands, on the Illumina NextSeq500 platform to obtain 76 bp paired-end reads. FASTQC and MultiQC (Ewels *et al*, 2016) software was used to assess and summarize the quality of the raw sequence data. Reads were aligned to the Mus musculus genome (primary assembly GRCm38, version 75) using GSNAP with (default) parameters (Wu and Nacu, 2010). Alignment was assessed using RSeQC (Wang *et al*, 2012), RNA-SeQC (DeLuca *et al*, 2012) and gene expression abundance (read count) was estimated. It was shown that estimation of gene expression is less reliable for genes with low read count (Soneson and Delorenzi, 2013) and therefore recommended to filter low-count genes prior to differential expression testing (Anders *et al*, 2013). Hence, transcripts with at least 10 reads in 5 samples were retained and further used to identify differentially expressed genes between the four experimental groups: water-GAS vs water-PBS, CORT-GAS vs water-GAS, CORT-PBS vs water-PBS, and CORT-GAS vs CORT-PBS. Statistical analyses were performed using R (www.r-project.org) and Bioconductor packages ((Huber *et al*, 2015), www.bioconductor.org), applying four methods: limma voom, limma voomWithQualityWeights (Ritchie *et al*, 2015), EdgeR glm (Robinson *et al*, 2010), (McCarthy *et al*, 2012) and DESeq2 (Love *et al*, 2014) to test for differences in gene expression between groups. Batch effects (library preparation, sequencing run, striatum hemisphere) were modeled according to the statistical design of each method. Genes were called as

differentially expressed if they passed the selection criteria threshold of absolute fold change ≥1.2 and P-value <0.01 for each of the four methods. The final sets of differentially expressed genes comprised those that were identified as differentially expressed by at least two of the four used methods. As suggested by Zhang et al. (Zhang *et al*, 2014), this approach was taken to account for false positive discoveries.

Venn diagrams (see Supplementary Figure 1) of the final sets of differentially expressed genes were built with Venny (Oliveros, 2007-2015). For genes that showed differential expression, upstream regulator analysis was performed using the Ingenuity Pathway Analysis software (IPA) (Ingenuity Systems Inc., Redwood City, CA). Based on the "Ingenuity Knowledge Base", a repository of data from publicly accessible databases and data that are manually curated by systematically reviewing published literature, IPA can generate a list of 'upstream regulators', i.e. proteins or compounds that regulate the expression of multiple target mRNAs/genes from an input list. The program also calculates a z-score that is based on the expression changes of the input mRNAs and that is a measure for the directionality of the upstream regulator. In this respect, a z-score <-1.5 or >1.5 (reflecting inhibition or activation of the upstream regulator-dependent effects on target gene expression, respectively) is considered as "suggestive".

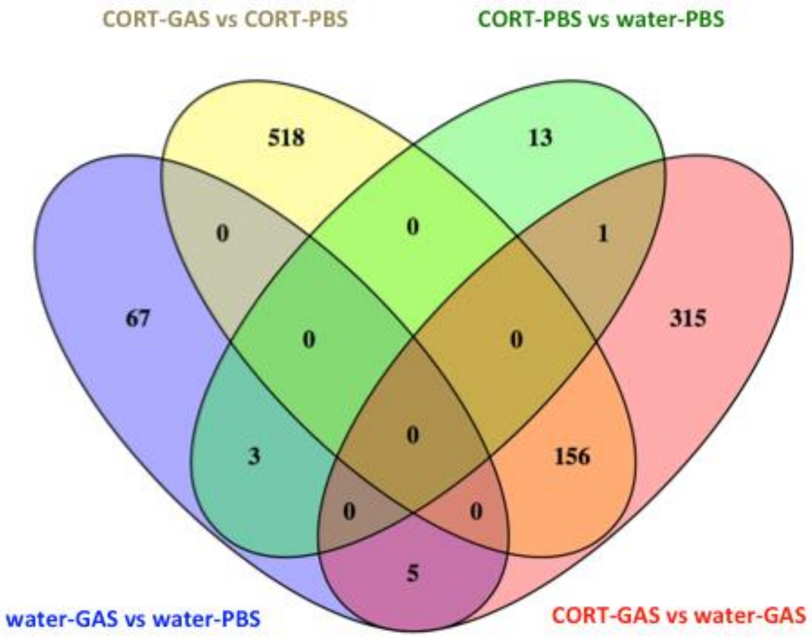

Supplementary Figure 1: Venn diagrams showing the unique and overlapping differentially expressed genes between the four comparisons, i.e. WATER-GAS vs WATER-PBS (75 genes), CORT-GAS vs WATER -GAS (477 genes), CORT-PBS vs WATER -PBS (17 genes), and CORT-GAS vs CORT-PBS (674 genes).

Supplementary Results and Tables

Antibody response to GAS administration

Predictably, while Western Blot analyses conducted on sera from WATER-GAS and CORT-GAS mice revealed several bands (Supplementary Fig. 2, lane 3 and 5 respectively), WATER-PBS and CORT-PBS mice did not reveal any or very few bands (Supplementary Fig. 2, lane 2 and 4 respectively).

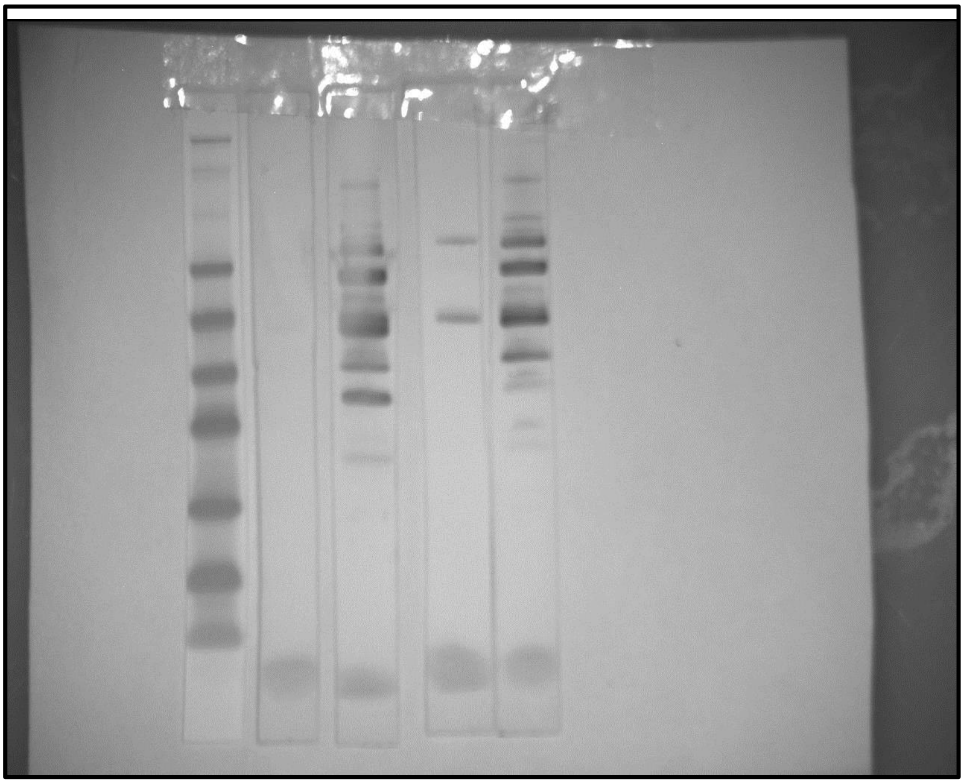

Supplementary figure 2: Western Blot analysis of GAS extracts probed with pooled sera from mice treated with GAS homogenates or adjuvant alone as Control, neonatal CORT and GAS homogenates or neonatal CORT and adjuvant. Lane 1, Coomassie staining of GAS homogenates; Lane 2, Western Blot results after probing with sera of control mice injected with four doses of adjuvant alone; lane 3, Western Blot results after probing with sera of mice injected with four doses of GAS homogenates; lane 4, Western Blot results after probing with sera of mice injected with four doses of neonatal CORT and adjuvant; lane 5, Western Blot results after probing with sera of mice injected with four doses of neonatal CORT and GAS homogenates.

Elevated 0-Maze (E0M) test (week 7, second injection)

As expected, all mice showed a preference for the closed sectors of the maze, wherein they spent on average 68,1% of the time. All experimental groups did not significantly differ in terms of time spent in the open arms (neonatal treatment: $F(1,34) = 1{,}395$, $p = 0{,}2457$; PBS/GAS treatment: $F(1,34) = 0{,}016$,

p = 0,9003; neonatal treatment x PBS/GAS treatment: *F*(1,34) = 0,614, *p* = 0,4388). The frequency of entries in the open sectors of the maze, considered an index of general locomotion, was significantly reduced in CORT-treated subjects (neonatal treatment: *F*(1,33) = 4,410, *p* = 0,0434) regardless of GAS administration (neonatal treatment x PBS/GAS treatment: *F*(1,33) = 0,021, *p* = 0,8866). Furthermore, GAS administration *per se* did not influence the frequency of open arm entries (PBS/GAS treatment: *F*(1,33) = 0,614, *p* = 0,4388). Finally, CORT-treated animals exhibited increased levels of *SAP* (duration, neonatal treatment: *F*(1,34) = 5,391, *p* = 0,0264) and decreased levels of *head dipping* (frequency, neonatal treatment: *F*(1,34) = 5,683, *p* = 0,0229; duration, neonatal treatment: *F*(1,34) = 6,797, *p* = 0,0135). None of the other behavioral parameters considered differed significantly among experimental groups (see Table 1, Supplementary Results).

Elevated Plus-Maze test (EPM, week 11, fourth injection)

We evaluated the behavioral profile of experimental subjects on the EPM after the fourth injection (week 11). As expected, mice showed an 85.9% preference (calculated as: 100 * time spent in the closed arms / (time in open arms + time in closed arms)) for the closed instead of the open arms of the maze. Experimental groups did not differ in terms of time spent in the open arms of the maze (neonatal treatment: *F*(1,34) = 0,076, *p* = 0,9217; PBS/GAS treatment: F(1,34) = 0,010, *p* = 0,7851; neonatal treatment x PBS/GAS treatment: F(1,34) = 0,492, *p* = 0,4877). Early CORT administration significantly reduced the time spent in the center of the apparatus (neonatal treatment: *F*(1,33) = 4,565, *p* = 0,0401). General locomotion did not significantly vary among groups (frequency of entries, neonatal treatment: *F*(1,34) = 0,711, *p* = 0,4050; PBS/GAS treatment: *F*(1,34) = 0,606, *p* = 0,4417; neonatal treatment x PBS/GAS treatment: *F*(1,34) = 0,092, *p* = 0,7640). With respect to the ethological parameters considered, we observed that early CORT administration significantly reduced *SAP* (frequency of *SAP*, neonatal treatment: *F*(1,33) = 4,621, *p* = 0,0390; duration of *SAP*, neonatal treatment: *F*(1,34) = 10,024, *p* = 0,0033) and *sniffing* (frequency of *sniffing*, neonatal treatment: *F*(1,34) = 6,227, *p* = 0,0176; duration of *sniffing*, neonatal treatment: *F*(1,34) = 4,132, *p* = 0,0499). We did not

observe between-group differences for all the other behavioral parameters considered (see Table 1, Supplementary Results).

Rotarod test (week 11, fourth injection)

We evaluated motor coordination through the Rotarod Test after the fourth injection (week 11). With respect to the latency to fall from the apparatus, all experimental subjects showed a progressive tendency towards an improvement of their performance across trials (trial: $F(2,68) = 7{,}410$, $p = 0{,}0012$). Additionally, the progressive improvement was undistinguishable among experimental groups (latency to fall, neonatal treatment: $F(1,34) = 0{,}806$; PBS/GAS treatment: $F(1,34) = 0{,}305$, $p = 0{,}5846$; neonatal treatment x PBS/GAS treatment: $F(1,34) = 0{,}467$, $p = 4988$).

T-maze (week 11, fourth injection)

Repeated GAS injections significantly reduced the percentage of spontaneous alternations in a T-maze test ($F(1,34) = 9{,}074$, $p = 0{,}0049$, see Supplementary Fig. 3). Neonatal corticosterone administration neither altered the exhibition of spontaneous alternations (neonatal treatment: $F(1,34) = 0{,}003$, $p = 0{,}9559$) nor influenced the consequences of repeated GAS administrations (neonatal treatment x PBS/GAS treatment: $F(1,34) = 1, 163$, $p = 0{,}2884$, see Supplementary Fig. 3).

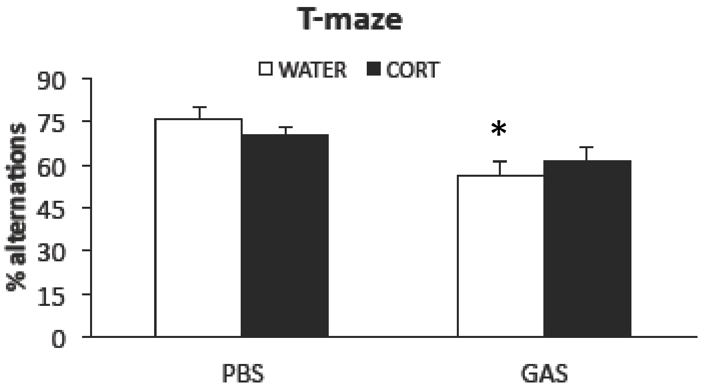

Supplementary figure 3: Perseverative behavior in a T-maze (week 11, fourth injection) measured as the percentage of spontaneous alternations (mean+SEM; n=8-12 per group). *p<0.05 in post-hoc tests versus WATER-PBS subjects.

Monoamine measurements

While striatal dopamine concentrations were apparently reduced in response to both treatments, such reduction was not statistically significant (neonatal treatment: $F(1,17) = 4{,}017$, $p = 0{,}0612$; PBS/GAS treatment: $F(1,17) = 4{,}058$, $p = 0{,}0601$).

RNA seq data analysis: effects at the molecular level

The upstream regulator analysis of the genes that were found to be differentially expressed in the striatum between the experimental groups (Table 1 and Supplementary Table 4) provides insights into the (putative) molecular mechanisms underlying the observed behavioral changes. More specifically, the analysis in Table 1 gives clues as to which mechanisms convey the effects of neonatal corticosterone treatment on GAS-induced abnormal behaviour.

First and foremost, the regulatory effect of (**beta-)estradiol** - the active form of the main female sex hormone estrogen that is converted from testosterone, the main male sex hormone, by the aromatase enzyme (The UniProt, 2017) - is predicted to be inhibited in GAS mice while it is activated in GAS mice that have been neonatally exposed to corticosterone (CORT) and that have attenuated GAS-induced behaviours. This is in keeping with literature findings, i.e. **estradiol**-deficient rodents show decreased PPI (Van den Buuse and Eikelis, 2001; van den Buuse *et al*, 2003) and develop OCD-like behavior (Flaisher-Grinberg *et al*, 2009; Hill *et al*, 2007; Mitra *et al*, 2016). In addition, tics have been reported to worsen in women during times of (relatively) lower **estradiol** levels (e.g. during the premenstrual phase) (Kompoliti *et al*, 2001; Schwabe and Konkol, 1992) and both in women and men - in whom estradiol can only be produced through conversion from testosterone - increased (and earlier) OCD symptoms have been linked to (relatively) lower **estradiol** levels (Boon and Horne, 2011; Martino *et al*, 2013). **Estradiol** has been reported to regulate the immune response through e.g. having a protective effect against bacterial infections (Garcia-Gomez *et al*, 2013). Interestingly, this effect is probably achieved through **estradiol** modulating the immunologic potency of **IgG** antibodies for that

are formed against bacteria (see below) (Ercan *et al*, 2017). Further, **estradiol** activates the HPA-axis, resulting in an increased **CORT** production and associated stress response (Bailey and Silver, 2014; Liu and Herbison, 2013; Mitsushima *et al*, 2008), reinforcing the observed attenuating effect of CORT on GAS-induced behavior. Lastly, **estradiol** is involved in upregulating the expression of **luteinizing hormone (LH)** (Turzillo *et al*, 1998) and it increases the striatal release of **dopamine (DA)** (Hussain *et al*, 2016; Shams *et al*, 2016) (see below).

As indicated above, testosterone is converted to beta-estradiol by the aromatase enzyme. Therefore, it is interesting that in GAS-induced mice, the effect of **dihydrotestosterone**, a metabolite that is converted from testosterone by 5α-reductase enzymes (The UniProt, 2017) of which one family member, SRD5A1, was found to be downregulated in GAS-induced mice (see Supplementary Table 4). **Dihydrotestosterone** also decreases the release of **LH** (Turgeon and Waring, 1999) and upregulates the expression of **LEP** (Gui *et al*, 2004) (see below).

The neurotransmitter **DA** is converted from its precursor, **L-dopa**, by the DDC enzyme (Gjedde *et al*, 1991; The UniProt, 2017). As already indicated above, the core hypothesis relating streptococcal infections to the onset of PANDAS is that **IgG** antibodies produced in response to GAS cross the blood brain barrier and are aimed at specific neuronal proteins (in the basal ganglia). In this respect, it was shown that **IgG** from GAS-exposed rats reacts with D1 and D2 **DA** receptors (D1R and D2R) in vitro. In vivo, **IgG** deposits in the striatum of infused rats co-localize with these dopamine receptors (Lotan *et al*, 2014). Further, **IgG** from human PANDAS patients was found to react with D2R and have a negative effect on signaling downstream of D2R (Cox *et al*, 2013). Individuals with tics and/or OCD also have elevated serum **IgG** against human D1R (Cox *et al*, 2015). In addition, it was demonstrated that **IgG** is sufficient to induce PANDAS symptoms in humans and the depletion of **IgG** from PANDAS patient sera alleviates these symptoms (Yaddanapudi *et al*, 2010). Therefore, it is interesting that **IgG**-dependent regulation is predicted to be inhibited in CORT-exposed GAS mice, which also fits with the reported negative effect of corticosteroids on **IgG** activity (Gill *et al*, 1999; Kurlander, 1981).

In CORT-exposed GAS mice, the downstream regulatory effects of **DA** are predicted to be activated while those of **L-dopa** are inhibited. This has to be considered against the findings from the literature on **DA/L-dopa** and tic disorders as well as OCD, i.e. both increased and decreased **DA** signaling have been suggested as etiological factors in these disorders. For example, decreased striatal **DA** receptor availability has been demonstrated in both tic disorders and OCD, presumably reflecting higher endogenous **DA** levels in both disorders (Denys *et al*, 2013). However, another study indicates that **DA** receptor availability is not different between tic disorder patients and controls (Abi-Jaoude *et al*, 2015).

Likewise, both **DA** agonists and antagonists - correcting for decreased and increased **DA** levels/signaling, respectively - have been reported to alleviate tics (Hershey *et al*, 2004). Moreover, **L-dopa** reduces tics (Black and Mink, 2000; Nomura and Segawa, 2003). In light of all these findings, it is difficult to correctly interpret the upstream regulator analysis results for **DA** and **L-dopa** in the CORT-exposed GAS mice so we can only conclude that **DA** signaling is dysregulated in GAS-induced mice. That being said, both **estradiol** (see above) and corticosterone (Piazza *et al*, 1996) increase striatal **DA** release, which is in line with the fact that **DA-**dependent regulation is predicted to be activated in CORT-exposed GAS mice.

LH is secreted by the pituitary gland upon stimulation by hypothalamic gonadotropin-release hormone (GnRH) and activates gonadal production of sex hormones (i.e. **estradiol**, testosterone, and progesterone), which then - as a part of the HPG axis - provide negative feedback to the hypothalamus (Martino *et al*, 2013). As already indicated above, **estradiol** upregulates the expression of **LH** while **dihydrotestosterone** decreases its secretion. In addition, **DA** - which is predicted to have an increased downstream effect in CORT-exposed GAS mice - is a potent *inhibitor* of GnRH-induced **LH** secretion by the pituitary (Grey and Chang, 2013; Liu *et al*, 2013). The latter finding seems to be in contradiction with the results of our experiments, i.e. that **LH**-dependent regulation is *activated* in CORT-exposed GAS mice. However, as mentioned before, it is not clear what the exact mechanisms are through which increased and/or decreased **DA** levels and activity alleviate the behavioral changes in CORT-exposed GAS mice.

Oleic acid-dependent regulation is inhibited in CORT-exposed GAS mice. In this respect, it is interesting that **oleic acid** is a fatty acid component of triglycerides (Igal *et al*, 2001) and increased triglyceride levels have been found in the serum of OCD patients (Agargun *et al*, 2004; Albert *et al*, 2013). In addition, **dihydrotestosterone** decreases the production of **oleic acid** (Fagman *et al*, 2015).

Interleukin-2 (IL2) is a cytokine that is crucial for regulating the T-cell-mediated immune response (The UniProt, 2017) and that is downregulated by **beta-estradiol** (McMurray *et al*, 2001). Further, **IgG-**mediated GAS infection leads to increased **IL2** expression in T cells (Mortensen *et al*, 2015).

IL2 decreases the release of **LH** from the anterior pituitary (Karanth and McCann, 1991), while high doses of **dopamine (DA)** (see below) reinforce the inhibitory effect of **IL2** on **LH** release (Karanth *et al*, 1992). Furthermore, **IL2**-dependent regulation is predicted to be inhibited in CORT-exposed GAS mice which is in line with the fact that an increased **IL2** expression was found postmortem in the basal ganglia of TS patients (Morer *et al*, 2010) and serum **IL2** concentrations were found to be positively associated with tic severity (Bos-Veneman *et al*, 2010). In addition, plasma levels of **IL2** were increased in drug-naive OCD patients (Rao *et al*, 2015). Lastly, chronic **IL2** exposure leads to an overactivation of the HPA axis, which in turn causes a significant increase in CORT plasma levels (Hanisch *et al*, 1994).

Fibroblast growth factor 2 (**FGF2**) has multiple roles, including the positive regulation of neurite outgrowth and, linked to this, motor function in rats (Wolf *et al*, 2014). **FGF2**-dependent regulation is predicted to be inhibited in GAS mice and interestingly, the effect of **FGF2** is activated when comparing CORT-GAS with CORT-PBS mice (see Supplementary Table 4). In addition, **estradiol** and **IL2** are involved in (up)regulating the expression of **FGF2** (Chaturvedi and Sarkar, 2004). Further, CORT exposure leads to an upregulation of **FGF2** in various brain regions, which fits with the observed inhibited **FGF2**-dependent regulation in GAS mice and may represent a defense mechanism through which the brain limits the deleterious effect of stress over time (Hansson *et al*, 2003; Magnaghi *et al*, 2000; Mocchetti *et al*, 1996; Molteni *et al*, 2001).

Leptin (LEP), a hormone and growth factor with multiple functions including appetite regulation (The UniProt, 2017), is predicted to be inhibited in the CORT-treated GAS mice, which fits with the finding that CORT downregulates **LEP** expression (Morash *et al*, 2000). Further, **dihydrotestosterone** and **beta-estradiol** are also involved in upregulating (Gui *et al*, 2004) and downregulating (Misso *et al*, 2003; Penza *et al*, 2006) **LEP** expression, respectively. Moreover, **LEP** inhibits the release of **DA**, which fits with the finding that in CORT-treated GAS mice, the effect of **LEP** is inhibited while **DA**-induced regulation is activated (Schwartz *et al*, 2000). Interestingly, **LEP** also plays a pro-inflammatory role through increasing **IL2** production by T-lymphocytes (Lord *et al*, 1998), which implies that the anti-inflammatory effect of CORT on GAS mice could be mediated in part by an inhibition of **LEP**-dependent regulation.

IKKbeta (IKBKB) is a cytoplasmic kinase that is predicted to be inhibited in CORT-exposed GAS mice and activates NF-kappaB (NF-κB), a pro-inflammatory transcription factor that is critical for the regulation of innate immunity (Lawrence *et al*, 2005). In this respect, it is interesting that an inducible deletion of ***Ikbkb*** reduces GAS infection in mice (Hsu *et al*, 2011) and that selective inactivation of ***Ikbkb*** normalizes OCD-like behavior in mice (Krabbe *et al*, 2017). Furthermore, both glucocorticoids and **estradiol** negatively regulate the activity of **IKBKB** (Simoncini *et al*, 2000). In addition, **IKBKB** is involved in upregulating the expression of **IL2** (Ren *et al*, 2002) while **FGF2** and **LEP** both regulate the activity of **IKBKB** (Lam *et al*, 2007; Vandermoere *et al*, 2005).

Lastly, the transmembrane protein **TREM-1** - which is highly expressed in neutrophils and macrophages - is a receptor for GAS and positively mediates the GAS-induced inflammatory response (Tsatsaronis *et al*, 2014). In this respect, it is interesting that **TREM-1** has been tested and confirmed as a drug target to block in the treatment of GAS-induced (Horst *et al*, 2013) and other immunity-related disorders (Murakami *et al*, 2009; Zhong *et al*, 2016). Further, it was demonstrated that **CORT** treatment significantly decreased the plasma expression levels of TREM-1 in febrile patients with autoimmune disease (Lin *et al*, 2016). This all fits very nicely with our findings, i.e. neonatal exposure to **CORT** led to an inhibition of **TREM-1**-dependent regulation in GAS mice.

Supplementary Table 1. Synopsis of the results obtained in the elevated zero-maze (A) and elevated plus-maze (B)

	WATER-PBS	WATER-GAS	CORT-PBS	CORT-GAS	Neonatal treatment		PBS/GAS treatment		Neonatal treatment x PBS/GAS treatment	
					F(1,34)	*p*	F(1,34)	*p*	F(1,34)	*p*
(A)										
Closed sectors(d)	201,74±9,40	195,2±11,3	205,7±9,48	214,69±8,96	1,397	0,2454	0,016	0,901	0,615	0,438
Closed sectors (f)	22,75±3,494	21±2,970	17,4±2,267	18,167±1,918	2,472	0,1252	0,036	0,851	0,234	0,632
SAP (d)	10,305±2,004	9,47±2,180	15,607±2,197	15,47±2,659	5,391	0,0264	0,040	0,843	0,021	0,887
Head dipping (f)	55±3,713	56±4,162	43,1±3,662	47,167±4,756	5,683	0,0229	0,339	0,564	0,124	0,727
Rearing (f)	17,5±2,885	14,5±1,427	16,5±2,478	18,417±1,454	0,473	0,4963	0,065	0,799	1,344	0,254
Grooming (d)	13,47±3,673	14,243±4,694	26,139±9,429	20,518±4,459	0,127	0,7241	0,089	0,768	0,018	0,895
Sniffing (f)	5,25±1,916	4±0,897	3,4±0,897	4,667±0,995	0,236	0,6299	0,00005	0,995	1,070	0,308
(B)										
Open arms (d)	36,1±8,999	29,86±9,584	28,5±7,37	33,17±5,69	0,076	0,7851	0,010	0,922	0,492	0,488
Open arms (f)	6,12±1,11	4,875±1,381	4,8±1,42	4,25±0,687	0,711	0,4050	0,606	0,442	0,092	0,764
Closed arms (d)	193±20,998	217,7±19,35	231,56±14,3	223,3±16,28	1,531	0,2244	0,211	0,649	0,855	0,362
Closed arms (f)	11,5±0,845	11±1,389	10±1,183	8,75±0,922	2,857	0,1001	0,622	0,436	0,114	0,737
% Open arms (f)	33,1±4,1	28,8±4,7	25,4±5,4	28,8±4,6	0,599	0,4445	0,008	0,929	0,621	0,436
SAP (d)	16,715±4,560	18,6±7,179	4,986±1,317	5,632±2,074	10,024	0,0033	0,105	0,748	0,025	0,875
Head dipping (f)	8,75±1,971	8,875±2,349	10,7±1,535	9,917±1,612	0,649	0,4259	0,031	0,860	0,060	0,808
Rearing (f)	12,75±1,634	13,625±1,238	14,1±1,574	12,917±1,401	0,045	0,8332	0,010	0,919	0,464	0,501
Grooming (d)	57,823±11,081	59,906±14,806	51,649±8,734	57,088±13,505	0,127	0,7241	0,089	0,768	0,018	0,895
Sniffing (f)	8,125±1,54	8,125±2,191	4,9±0,849	4,75±0,789	6,227	0,0176	0,003	0,955	0,003	0,955

Behavioral phenotypes (mean±SEM; N=38) exhibited by adolescent (A) and adult (B) WATER-PBS (n=8), WATER-GAS (n=8), CORT-PBS (n=10) and CORT-GAS (n=12) mice. The three rightmost columns indicate the F and p values (together with degrees of freedom) of the main effects of neonatal treatment, PBS/GAS treatment, and their interaction.

Supplementary table 2. Monoamine concentrations in selected brain areas.

	WATER-PBS	WATER-GAS	CORT-PBS	CORT-GAS	Neonatal treatment		PBS/GAS treatment		Neonatal treatment x PBS/GAS treatment	
					F(1,21)	*p*	F(1,21)	*p*	F(1,21)	*p*
(A)										
DA	30.200±12.142	24.333±5.064	58.143±21.835	25.286±3.441	1.109	0.163	1.991	0.1729	0.967	0.3365
DOPAC	69.200±30.170	42.333±3.703	57.714±18.855	45.714±10.936	0.055	0.8171	1.261	0.2742	0.184	0.6719
DOPAC/DA	2.940±0.780	2.793±1.103	1.516±0.289	1.832±0.301	3.268	0.0850	0.016	0.8995	0.124	0.7286
5-HT	455±69.804	396±84.367	585±95.201	503±55.012	2.208	0.1521	1.991	0.1729	0.021	0.8867
5-HIAA	261.400±41.086	209.167±19.919	279.857±55.183	232.143±38.420	0.235	0.6331	1.365	0.2557	0.003	0.9584
5-HIAA/5-HT	0.593±0.070	0.615±0.087	0.503±0.060	0.485±0.071	2.263	0.1474	0.001	0.9748	0.074	0.7889
(B)										
DA	13391.5±2077.654	11180.6±1167.124	11192.667±537.17	9150.667±1082.517	4.017	0.0612	4.058	0.0601	0.089	0.7694
DOPAC	2471.75±370.522	1784.167±142.165	1784.167±201.933	1566±160.949	12.073	0.0029	4.456	0.0499	1.155	0.2976
DOPAC/DA	0.200±0.020	0.190±0.021	0.160±0.012	0.177±0.021	1.856	0.1909	0.026	0.8744	0.534	0.4748
5-HT	473.250±62.073	355.800±56.840	328.500±18.938	277±30.670	7.239	0.0155	4.134	0.0579	0.630	0.4383
5-HIAA	338.500±33.977	259.200±28.645	245.333±15.322	210.333±17.718	9.370	0.0071	6.068	0.0247	0.912	0.3531
5-HIAA/5-HT	0.729±0.039	0.753±0.053	0.748±0.028	0.784±0.069	0.231	0.6366	0.323	0.5775	0.014	0.9088
(C)										
DA	43.600±25.789	234.667±202.316	36.857±9.674	28.714±7.846	1.167	0.2923	0.863	0.3634	1.024	0.3231
DOPAC	65±30.158	147±89.506	53.143±12.219	40.714±8.983	1.675	0.2096	0.581	0.4544	1.070	0.3127
DOPAC/DA	1.809±0.166	1.823±0.322	1.614±0.182	1.703±0.233	0.435	0.5165	0.046	0.8319	0.025	0.8763
5-HT	429.8±42.878	422.5±75.996	469.857±65.376	464.429±38.860	0.478	0.4971	0.012	0.9156	0.0003	0.9876
5-HIAA	568.2±57.504	511.500±86.119	592.571±88.826	600.714±45.327	0.588	0.4517	0.107	0.7463	0.192	0.6660
5-HIAA/5-HT	1.327±0.061	1.281±0.174	1.264±0.080	1.329±0.095	0.012	0.9136	0.002	0.9681	0.199	0.6598
(D)										
DA	128.250±36.346	260.167±29.703	297.286±43.340	221.286±24.610	3.289	0.0848	0.607	0.4450	8.394	0.0089[€]
DOPAC	114.750±29.304	160±13.140	181.857±28.117	157.857±26.934	1.518	0.2322	0.162	0.6912	1.725	0.2040
DOPAC/DA	1.032±0.169	0.633±0.041	0.616±0.035	0.692±0.078	5.184	0.0339	4.225	0.0531	9.180	0.0066[°]
5-HT	593.250±175.001	664.667±72.203	795.429±47.884	810.429±63.982	4.352	0.500	0.268	0.6101	0.114	0.7387
5-HIAA	480.500±132.751	432.500±19.419	501.714±26.225	501.286±62.472	0.555	0.4650	0.161	0.6928	0.155	0.6979
5-HIAA/5-HT	0.848±0.050	0.684±0.065	0.638±0.033	0.618±0.068	6.399	0.0199	2.823	0.1085	1.718	0.2048

Concentrations of: dopamine (DA), its metabolite 3,4-dihydroxyphenylacetic acid (DOPAC) and turnover (DOPAC/DA); and serotonin (5-HT), its metabolite 5-hydroxyndole acetic acid (5-HIAA) and turnover (5-HIAA/5-HT). Data, expressed as mean±SEM (N=25), refer to prefrontal cortex (A), striatum (B), hippocampus (C) and hypothalamus (D) of adult WATER-PBS (n=5), WATER-GAS (n=6), CORT-PBS (n=7) and CORT-GAS (n=7) mice (ng/g tissue weight). The three rightmost columns indicate the F and p values (together with degrees of freedom) of the main effects of Neonatal treatment, PBS/GAS treatment, and their interaction. [€] Post-hoc comparisons conducted based on this significant interaction show that CORT-GAS mice have increased concentrations of DA compared to WATER-PBS subjects. [°] Post-hoc comparisons conducted based on this significant interaction show that CORT-GAS mice have reduced concentrations of dopamine turnover (DOPAC/DA) compared to WATER-PBS subjects.

Supplementary table 3. Cytokines concentrations measured through Bio-Plex assay

	WATER-PBS	WATER-GAS	CORT-PBS	CORT-GAS	Neonatal treatment		PBS/GAS treatment		Neonatal treatment x PBS/GAS treatment	
					F(1,33)	*p*	F(1,33)	*p*	F(1,33)	*p*
IL-1α	22.924±7.551	18.720±5.547	25.987±6.183	28.592±6.169	0.993	0.3264	0.015	0.9027	0.275	0.6033
IL-1β	140.088±74.173	47.265±26.270	160.029±49.587	168.805±44.508	1.838	0.1846	0.649	0.4265	0.948	0.3375
IL-2	4.443±2.683	5.271±3.511	2.893±0.881	5.164±1.946	0.124	0.7272	0.434	0.5156	0.094	0.7614
IL-4	26.643±12.070	10.273±3.707	31.714±8.736	30.882±7.560	2.132	0.1540	0.956	0.3354	0.780	0.3836
IL-5	60.094±23.792	48.368±16.569	73.099±16.834	73.107±14.827	1.112	0.2992	0.107	0.7454	0.108	0.7451
IL-6	16.833±9.181	10.161±6.265	23.711±7.686	21.881±7.003	1.433	0.2382	0.302	0.5866	0.098	0.7564
IL-9	29.964±18.625	20.247±18.572	151.221±51.374	40.778±17.479	4.717	0.0372	3.387	0.0747	2.380	0.1324
IL-10	26.951±13.666	21.156±9.635	22.490±6.961	40.547±11.507	0.437	0.5137	0.295	0.5912	1.114	0.2993
IL-12 (p40)	183.868±16.774	269.915±39.717	256.023±25.159	235.985±18.784	0.686	0.4140	1.168	0.2884	3.294	0.0796
IL-12 (p70)	171.641±92.267	99.930±43.821	133.633±44.496	201.708±74.426	0.213	0.6479	0.001	0.9792	1.022	0.3201
IL-13	300.687±152.826	125.080±50.982	389.125±118.941	405.037±99.229	2.528	0.1217	0.475	0.4957	0.683	0.4146
IL-17	97.993±32.558	78.069±20.155	176.910±43.508	153.938±38.384	5.094	0.0307	0.391	0.5359	0.002	0.9648
IFN-γ	30.026±15.311	17.267±8.608	41.824±13.003	46.605±12.798	2.318	0.1374	0.116	0.7356	0.394	0.5347
KC	35.112±12.111	28.484±6.347	47.963±7.517	49.807±10.556	3.028	0.0912	0.059	0.8091	0.186	0.6690
MCP-1	278.820±128.265	182.994±58.424	496.364±132.571	547.354±130.244	4.778	0.0365	0.028	0.8674	0.304	0.5853
MIP-1β	33.355±16.086	20.819±6.079	69.099±18.159	67.513±17.701	5.320	0.0279	0.156	0.6954	0.094	0.7614
RANTES	8.711±2.270	6.565±1.190	9.134±1.633	12.700±2.260	2.268	0.1141	0.124	0.7270	2.010	0.1666
TNF-α	213.822±128.008	123.126±56.475	377.411±126.426	585.178±176.747	3.949	0.0561	0.138	0.7126	0.899	0.3507

Plasma concentrations (mean±SEM; N=37), in serum of adult WATER-PBS (n=7), WATER-GAS (n=8), CORT-PBS (n=10) and CORT-GAS (n=12) mice (pg/ml) of the following cytokines: interleukin 1α (IL-1α), interleukin 1β (IL-1β), interleukin 2 (IL-2), interleukin 4 (IL-4), interleukin 6 (IL-6), interleukin 9 (IL-9), interleukin 10 (IL-10), interleukin 12 (p40) (IL-12 (p40)), interleukin 12 p70 (IL-12 (p70)), interleukin 13 (IL-13), interleukin17 (IL-17), interferon γ (IFN-γ), keratinocyte chemoattractant (KC), monocyte chemoattractant protein-1 (MCP-1), macrophage inflammatory protein 1β (MIP-1β), Regulated on Activation, Normal T Cell Expressed and Secreted (RANTES), Tumor necrosis factor α (TNF-α). The three rightmost columns indicate the F and p values (together with degrees of freedom) of the main effects of Neonatal treatment, PBS/GAS treatment, and their interaction.

Supplementary Table 4. Upstream regulator analysis - using Ingenuity - of the mRNAs that were differentially expressed in GAS-treated mice compared to controls (water-GAS vs water-PBS) (1), GAS-treated mice neonatally exposed to CORT compared to GAS-treated mice (CORT-GAS vs water-GAS) (2), CORT-exposed mice compared to control (CORT-PBS vs water-PBS) and GAS-treated mice neonatally exposed to CORT compared to CORT-exposed mice (CORT-GAS vs CORT-PBS). All upstream regulators are listed with a z-score ≥ 1,50 or ≤ - 1,50 (see Methods), indicating that they are activated or inhibited, respectively. For each regulator, the downstream target genes are listed.

Upstream Regulator	water-GAS vs water-PBS (1)	CORT-GAS vs water-GAS (2)	CORT-PBS vs water-PBS (3)	CORT-GAS vs CORT-PBS (4)	Target genes
Chemicals - endogenous mammalian					
beta-estradiol	-1,97	2,47	-	2,58	(1): Btg2, Cbl, Cshl1, Enpp2, Fos, Inpp5j, Sdc3, Sema3b, Srd5a1, Tnnt3; (2): Adm, Bcat1, Bmp2, Calb1, Ccng1, Cdkn2b, Chgb, Ctps1, Dlg2, Fgf9, Fmo1, Fnbp1, Fzd2, Gab2, Gsk3b, Hsd17b12, Ier2, Insl3, Lhcgr, Lmcd1, Mapt, Mpz, Myh3, Nedd4l, Nkx2-1, Notch3, Pak5, Pdap1, Pitpna, Ptgds, Pttg1, Pxdn, Pycr1, Pygl, Qsox1, Rapgef6, Slc38a2, Smad3, Snap25, Sstr4, Tgfb3, Thrsp, Timp3, Tpo, Trib1, Vav3, Zyx; (4): Ace, Acta2, Adora1, Alcam, Aldh1a2, Blnk, Bmp2, C8orf44-SGK3/SGK3, Calb1, Casp9, Cav1, Ccl2, Ccne1, Cdc25a, Cdkn2b, Chgb, Chrm3, Clstn2, Clybl, Col1a2, Ctps1, Cyth3, Dcn, Dyrk2, Etv4, Fhl2, Fmo1, Gadd45a, Grik4, Grin2a, Gucy1b3, Hdac4, Hes1, Hunk, Id4, Ifngr2, Il18, Itgb3, Jag1, Klf10, Klhl26, Ldha, Ltbp1, Mapt, Mpz, Myh3, Nell2, Net1, Nptx1, Nrp1, Nucb2, Pak5, Pdgfb, Pdk2, Pik3r2, Pitpna, Plau, Pmm2, Ppp5c, Prss23, Prss35, Ptger2, Rab6a, Rara, Rdh10, Runx2, Sdc2, Serpina3, Sertad4, Snai1, Snap25, Spock1, Sstr4, Stc1, Thrsp, Timp3, Tpd52l1, Trib1, Tspan5, Ttr, Vav3, Ypel3, Ywhah, Zbtb18, Zeb2, Zyx
butyric acid	-	2,16	-	-	Calb1, Ccng1, Col5a2, Gli3, Ly6e, Myh3, Nfatc4, Oas2, Ptgds, Pygl, Serpine1, Timp3, Uqcrq
dihydrotestosterone	-1,95	-	-	-	Btg2, Elovl7, Fos, Sdc3, Slc7a7, Srd5a1
dopamine		1,63		2,00	(2): Adra2c, Bcl2l2, Gprc5b, Ncf1, Ppp2r2a, Scn1b, Scn9a; (4): Asic2, Bcl2l2, Gpr68, Ppp1r2, Scn1b
L-dopa	-	-4,59	-	-4,62	(2): Adra2c, Ajap1, C1qtnf12, C2cd2l, Cbr3, Cdc42ep2, Csmd3, Cyld, Dgki, Dlg2, Grid2, Kcne5, Klf16, Mpp6, Nedd4l, Per2, Plekha2, Ppp2r2a, Ptprd, Rell1, Reln, Slc38a2, Sorcs2, Wdr17, Zfhx3; (4): Acta2, Ai987944 (Includes Others), C14orf37, Clic6, Crtac1, Cyld, Dlgap1, Erlin1, Htr1b, Lrrc1, Ltbp1, Mterf2, Myl4, Nos1ap, Plekha2, Plppr1, Ppm1l, Ppp1r2, Prickle1, Rapgefl1, Rassf5, Rell1, Spock3, Tcf4, Tmem238, Trib3, Vat1l, Wdr1, Zeb2
oleic acid	-	-2,00	-	-	Ncf1, Npc1l1, Serpine1, Thrsp
progesterone	-	-	-	-1,96	Ace, Acta2, Aldh1a2, Bmp2, Cav1, Ccl2, Ccne1, Cdc25a, Cdkn2b, Dnajb4, Dpp4, Hunk, Klf10, Ldha, Mpz, Net1, Npl, Nrp1, P2rx4, Pnkd, Prss23, Prss35, Ptger1, Ptger2, Rbp1, Sfi1, Spsb1, Thrsp, Timp3, Tnfrsf21, Ttr
tretinoin	-	-	-	2,53	Adam11, Aldh1a2, Ap1s2, Bmp2, Calb1, Casp9, Ccl2, Ccne1, Cd34, Cd59a, Cdkn2b, Chgb, Col1a2, Csrnp1, Cyp2s1, Dcn, Dkk3, Dpp4, E2f5, Fgf5, Gtpbp10, Hes1, Hey2, Hgs, Hk1, Hmga1, Ifit1, Ighm, Itgb3, Kctd11, Klf13, Limk1, Ly6e, Mapk8ip3, Mgst3, Nid2, Nrp1, Nthl1, P4ha2, Pdgfb, Plau, Plk3, Prtn3, Psmb10, Rara, Rbp1, Runx2, Serpinb8, Slco2a1, Stc1, Wnt2b, Zbtb16, Zeb2
Complexes					
IgG	-	-2,24	-	-	Adm, Cdkn2b, Csnk2b, Ier2, Serpine1
LH	-	2,00	-	-	Actn1, Btc, Gprc5b, Insl3, Lhcgr, Snap25, Thbs2, Trib1
PDGF	-	-	-	1,80	Acta2, Anxa11, Ccl2, Ccne1, Dcn, Fbln5, Fhl2, Fmo1, Gadd45a, Gucy1b3, Hdac4, Hes1, Klf10, Klhl21, Myh11, Plau, Prss35, Rbp1, Serpina3, Snai1, Trib1, Trib3
Cytokines					
IL1A	-	-	-	1,50	Asic2, Ccl2, Ccr6, Dpp4, Ifngr2, Il18, Itgb3, Ldha, Lox, Pdgfb, Plau, Serpina3
IL2	-	-1,76	-		Bmp2, Card9, Ccng1, Ccr6, Cmklr1, Csf2rb, Csrnp1, Ctps1, Dapk2, Gcnt1, Ly6e, Pus1, Tnfrsf4
SPP1	-	-	-	1,66	Ccl2, Ccne1, Cd163, Cdc25a, Itgb3, Jag1, Lox, Plau, Runx2, Snai1
TNF	-	-	-	2,26	Acan, Ace, Acta2, Adora1, Alcam, Anxa11, Arhgdib, Bcl2l2, Bmp2, Cav1, Ccl2, Ccne1, Ccr6, Cd163, Col1a2, Cyld, Cyth3, Dcn, Dpp4, Edar, Ext1, Fgf5, Fmo1, Gadd45a, Gast, Hes1, Hid1, Ifit1, Ifngr2, Il18, Itgb3, Jag1, Klf10, Ldha, Lox, Map2k4, Net1, Nrp1, Nucb2, Pdgfb, Plau, Plk3, Ppp2r1b, Ppp5c, Prss23, Prtn3, Psmb10, Rab6a, Rapgefl1, Rara, Rbp1, Runx2, Sdc2, Serpina3, Serpinb8, Sirt3, Snai1, Spock1, Spsb1, St8sia4, Tcfl5, Thrsp, Timp3, Tnfrsf21, Vash1, Vav3, Wisp1, Zyx
WNT1	-	-	-	1,76	Acta2, Etv4, Hes1, Ly6e, Runx2, Wisp1
Enzymes					
CASZ1	-	-1,96	-	-	Acan, Myh3, Nefl, Tgfb3
EGLN1	-	-1,98	-	-	Acan, Adm, Cdkn2b, Tgfb3
TGM2	-	1,91	-	2,28	(2): Acan, Csrnp1, Dapk2, Ifit2, Ly6e, Mpp6, Oas2; (4): Acan, Ccl2, Csrnp1, E2f5, Gtpbp10, Ifit1, Itgb3, Ly6e, Rara, Runx2

**Supplementary Table 4 - continued**					
Upstream Regulator	water-GAS vs water-PBS (1)	CORT-GAS vs water-GAS (2)	CORT-PBS vs water-PBS (3)	CORT-GAS vs CORT-PBS (4)	Target genes
G-Protein Coupled Receptors					
ADORA2A	-	-	-	2,24	*Dgkz, Ifitm3, Klc1, Rab6a, Tubb4a*
Growth factors					
BDNF	-	-	-	2,29	*Bcl2l2, Bmp2, Crmp1, Grin2a, Klc1, Klf10, Limk1, Mapk8ip3, Mapt, Myh3, Myl4, Nell2, Snap25, Sorl1*
BMP7	-	-	-	1,94	*Acta2, Adora1, Aldh1a2, Ccl2, Cd34, Col1a2, Runx2*
DKK1	-	-	-	-1,98	*Acta2, Dvl3, Runx2, Tcf4*
EGF	-	-	-	2,56	*Alcam, Cav1, Ccne1, Chgb, Col1a2, Dcn, Dpp4, Gadd45a, Gast, Hes1, Itgb3, Kctd11, Klf10, Ldha, Nrp1, Plau, Runx2, Serpina3, Snai1, Snap25, Timp3*
FGF2	-1,93	-	-	1,90	*(1): Btg2, Enpp2, Fos, Prkce, Ptma (Includes Others); (4): Ace, Acta2, Calb1, Cav1, Ccl2, Cdc25a, Chrm3, Cnmd, Col1a2, Dcn, Gadd45a, Grik4, Grin2a, Lox, Mapt, Mpz, Pdgfb, Plau, Runx2, Snai1, Timp3*
INHBA	-	-2,34	-	-	*Actn1, Bmp2, Calb1, Cdkn2b, Edar, Lhcgr, Nkx2-1, Pcdh9, Serpine1, Vav3*
LEP	-	-2,16	-	-	*Cps1, Dffa, Gsk3b, Insl3, Ncf1, Per2, Pln, Rfx1, Serpine1, Snap25, Thrsp, Timp3*
NGF	-	-	-	2,11	*Asic2, Cav1, Chgb, Etv4, Gadd45a, Grin2a, Gucy1b3, Kctd11, Mapk8ip3, Snap25*
NRG1	-	-	-	2,08	*Cav1, Ccne1, Col6a3, Hes1, Hmga1, Itgb3, Plau, Snai1*
TGFB1	-	-	-	1,88	*Acan, Ace, Acta2, Adam19, Adamts3, Adora1, Anxa11, Bmp2, C1r, Casp9, Cav1, Ccl2, Ccne1, Ccr6, Cd163, Cd34, Cdc25a, Cdkn2b, Col1a2, Col6a3, Crmp1, Ctps1, Dach1, Dcn, Dkk3, Dnajb4, Dyrk2, Ext1, Fbln5, Fgf5, Gabra4, Gadd45a, Gpr146, Grin2a, Hes1, Hmga1, Id4, Ighm, Il18, Itgb3, Jag1, Klf10, Ldha, Lox, Ltbp1, Mapk8ip3, Masp1, Mpz, Myh11, Net1, Nfib, Nrp1, Nucb2, Pdgfb, Pdk2, Pigf, Plau, Pmm1, Prkcg, Ptger2, Rab6a, Rara, Rasl11b, Rbms1, Runx2, Serpina3, Snai1, Spock1, Tcf4, Timp3, Vat1l, Wisp1, Xylt1, Zeb2, Zfpm2, Zyx*
VEGFA	-	-	-	1,68	*Ace, Cav1, Ccl2, Ccne1, Cd34, Etv4, Hes1, Hk1, Itgb3, Me2, Nrp1, Pdgfb, Plau, Runx2, Stc1, Uqcrb, Vash1*
Kinases					
ERBB2	-			2,00	*Abracl, Acta2, Adam19, Ccne1, Cd34, Cdc25a, Cdkn2b, Col6a3, E2f5, Etv4, Hes1, Itgb3, Jag1, Mapk8ip3, Net1, Nfib, Nrp1, Nucb2, P4ha2, Plau, Poldip2, Serpina3, Snai1, Spock1, Timp3, Tpd52l1, Tubb4a*
IKBKB	-	-1,98			*Bmp2, Cdh13, Timp3, Tnfrsf4*
MAPK1	-	-	-	-1,60	*Cav1, Ccl2, Cetn4, Dxo, Fam20a, Fhl2, Ifit1, Ifitm3, Itgb3, Nrp1, Pdgfb, Plau, Ptger2, Snai1, Spock1, Spsb1, Ube2i*
MKNK1	-	-	-	2,65	*Crmp1, Klc1, Klf13, Mapk8ip3, Mapt, Nell2, Snap25*
PRKAA2	-	-	-	-1,54	*Acta2, Arhgdib, Chgb, Ldha, Nfib, Pak5, Snai1, Sorl1, Zyx*
Ligand-Dependent Nuclear Receptors					
PGR	-	-	-	2,20	*Dnajb4, Hes1, Net1, P2rx4, P4ha2, Plau, Serpinb8, Sfi1, Snap25, Stc1, Tnfrsf21*
Transcriptional Regulators					
ASCL1	-	-	-	2,00	*Ephb3, Hey2, Nfasc, Snap25*
BRCA1	-	-	-	2,35	*Atp11c, Ccne1, Dgka, Ensa, Fhl2, Gadd45a, Ifit1, Stc1, Ube2i*
CTNNB1	-	-	-	1,58	*Acan, Acta2, Alcam, Aldh1a2, Bmp2, C14orf37, Ccne1, Cd34, Cdkn2b, Cnmd, Dnajc6, Ephb3, Etv4, Fgf5, Gast, Hes1, Id4, Ifit1, Ighm, Ly6e, Mpz, Myh3, Myl4, Nptx1, Pcdh9, Plau, Prss35, Rassf5, Rcn1, Runx2, Sdc2, Sema5a, Serpina3, Snai1, Tcf4, Timp3, Wisp1, Ypel3, Zeb2*
EHF	-	-	-	2,00	*Blnk, Gpr68, Jag1, Serpina3, Tcf4*
FOS	-	-	-	2,80	*Ccl2, Ccne1, Large1, Lox, Ltbp1, Net1, Nptx1, Plau, Rara, Rbp1, Snai1, Sulf2, Sult2b1, Vav3, Xylt1*
GATA4	-	-	-	2,21	*Adora1, Cdkn2b, Clstn2, Col1a2, Myl4*
HIC1	-	-	-	-2,00	*Acta2, Fhl2, Id4, Snap25*
JUN	-	-	-	1,85	*Cav1, Ccl2, Col1a2, Fbln5, Hes1, Hmga1, Ltbp1, Mpz, Nrap, Plau, Rara, Runx2, Sulf2, Vav3, Xylt1*
JUNB	-	-	-	1,73	*Cav1, Col1a2, Fbln5, Hes1, Itgb3, Plau, Runx2, Snai1*
KDM5B	-	-	-	2,00	*Cav1, Ccne1, Cdc14a, Gadd45a, Kif2c, Mapk8ip3*
KLF2	-	-	-	-1,77	*Ace, Cav1, Ccl2, Fgf5, Gadd45a, Itgb3, Runx2, Slco2a1, Tcf4*
KMT2A	-	2,00	-		*Gcnt4, Pigz, Trib1, Zmat4*
MEF2C	-	-	-	1,97	*Clstn2, Col1a2, Myl4, Runx2*

References

Andrews S. 2015. FastQC: A Quality Control Tool for High Throughput Sequence Data. Available from: http://www.bioinformatics.babraham.ac.uk/projects/fastqc.

Abi-Jaoude E, Segura B, Obeso I, Cho SS, Houle S, Lang AE, *et al* (2015). Similar striatal D2/D3 dopamine receptor availability in adults with Tourette syndrome compared with healthy controls: A [(11) C]-(+)-PHNO and [(11) C]raclopride positron emission tomography imaging study. *Hum Brain Mapp* **36**(7): 2592-2601.

Agargun MY, Dulger H, Inci R, Kara H, Ozer OA, Sekeroglu MR, *et al* (2004). Serum lipid concentrations in obsessive-compulsive disorder patients with and without panic attacks. *Can J Psychiatry* **49**(11): 776-778.

Albert U, Aguglia A, Chiarle A, Bogetto F, Maina G (2013). Metabolic syndrome and obsessive-compulsive disorder: a naturalistic Italian study. *Gen Hosp Psychiatry* **35**(2): 154-159.

Anders S, McCarthy DJ, Chen Y, Okoniewski M, Smyth GK, Huber W, *et al* (2013). Count-based differential expression analysis of RNA sequencing data using R and Bioconductor. *Nat Protoc* **8**(9): 1765-1786.

Bailey M, Silver R (2014). Sex differences in circadian timing systems: implications for disease. *Front Neuroendocrinol* **35**(1): 111-139.

Black KJ, Mink JW (2000). Response to levodopa challenge in Tourette syndrome. *Mov Disord* **15**(6): 1194-1198.

Boon WC, Horne MK (2011). Aromatase and its inhibition in behaviour, obsessive compulsive disorder and parkinsonism. *Steroids* **76**(8): 816-819.

Bos-Veneman NG, Bijzet J, Limburg PC, Minderaa RB, Kallenberg CG, Hoekstra PJ (2010). Cytokines and soluble adhesion molecules in children and adolescents with a tic disorder. *Prog Neuropsychopharmacol Biol Psychiatry* **34**(8): 1390-1395.

Canal CE, Morgan D (2012). Head-twitch response in rodents induced by the hallucinogen 2,5-dimethoxy-4-iodoamphetamine: a comprehensive history, a re-evaluation of mechanisms, and its utility as a model. *Drug Test Anal* **4**(7-8): 556-576.

Chaturvedi K, Sarkar DK (2004). Involvement of protein kinase C-dependent mitogen-activated protein kinase p44/42 signaling pathway for cross-talk between estradiol and transforming growth factor-beta3 in increasing basic fibroblast growth factor in folliculostellate cells. *Endocrinology* **145**(2): 706-715.

Cox CJ, Sharma M, Leckman JF, Zuccolo J, Zuccolo A, Kovoor A, *et al* (2013). Brain human monoclonal autoantibody from sydenham chorea targets dopaminergic neurons in transgenic mice and signals dopamine D2 receptor: implications in human disease. *J Immunol* **191**(11): 5524-5541.

Cox CJ, Zuccolo AJ, Edwards EV, Mascaro-Blanco A, Alvarez K, Stoner J, *et al* (2015). Antineuronal antibodies in a heterogeneous group of youth and young adults with tics and obsessive-compulsive disorder. *J Child Adolesc Psychopharmacol* **25**(1): 76-85.

Deacon RM, Rawlins JN (2006). T-maze alternation in the rodent. *Nat Protoc* **1**(1): 7-12.

Dell'Omo G, Vannoni E, Vyssotski AL, Di Bari MA, Nonno R, Agrimi U*, et al* (2002). Early behavioural changes in mice infected with BSE and scrapie: automated home cage monitoring reveals prion strain differences. *Eur J Neurosci* **16**(4): 735-742.

DeLuca DS, Levin JZ, Sivachenko A, Fennell T, Nazaire MD, Williams C*, et al* (2012). RNA-SeQC: RNA-seq metrics for quality control and process optimization. *Bioinformatics* **28**(11): 1530-1532.

Denys D, de Vries F, Cath D, Figee M, Vulink N, Veltman DJ*, et al* (2013). Dopaminergic activity in Tourette syndrome and obsessive-compulsive disorder. *Eur Neuropsychopharmacol* **23**(11): 1423-1431.

Egashira N, Shirakawa A, Okuno R, Mishima K, Iwasaki K, Oishi R*, et al* (2011). Role of endocannabinoid and glutamatergic systems in DOI-induced head-twitch response in mice. *Pharmacol Biochem Behav* **99**(1): 52-58.

Ercan A, Kohrt WM, Cui J, Deane KD, Pezer M, Yu EW*, et al* (2017). Estrogens regulate glycosylation of IgG in women and men. *JCI Insight* **2**(4): e89703.

Ewels P, Magnusson M, Lundin S, Kaller M (2016). MultiQC: summarize analysis results for multiple tools and samples in a single report. *Bioinformatics* **32**(19): 3047-3048.

Fagman JB, Wilhelmson AS, Motta BM, Pirazzi C, Alexanderson C, De Gendt K*, et al* (2015). The androgen receptor confers protection against diet-induced atherosclerosis, obesity, and dyslipidemia in female mice. *Faseb j* **29**(4): 1540-1550.

Flaisher-Grinberg S, Albelda N, Gitter L, Weltman K, Arad M, Joel D (2009). Ovarian hormones modulate 'compulsive' lever-pressing in female rats. *Horm Behav* **55**(2): 356-365.

Flies DB, Chen L (2003). A simple and rapid vortex method for preparing antigen/adjuvant emulsions for immunization. *J Immunol Methods* **276**(1-2): 239-242.

Gao W, Stalder T, Foley P, Rauh M, Deng H, Kirschbaum C (2013). Quantitative analysis of steroid hormones in human hair using a column-switching LC-APCI-MS/MS assay. *J Chromatogr B Analyt Technol Biomed Life Sci* **928**: 1-8.

Garcia-Gomez E, Gonzalez-Pedrajo B, Camacho-Arroyo I (2013). Role of sex steroid hormones in bacterial-host interactions. *Biomed Res Int* **2013**: 928290.

Gill RK, Mahmood S, Sodhi CP, Nagpaul JP, Mahmood A (1999). IgG binding and expression of its receptor in rat intestine during postnatal development. *Indian J Biochem Biophys* **36**(4): 252-257.

Gjedde A, Reith J, Dyve S, Leger G, Guttman M, Diksic M*, et al* (1991). Dopa decarboxylase activity of the living human brain. *Proc Natl Acad Sci U S A* **88**(7): 2721-2725.

Grey CL, Chang JP (2013). Differential modulation of ghrelin-induced GH and LH release by PACAP and dopamine in goldfish pituitary cells. *Gen Comp Endocrinol* **191**: 215-224.

Gui Y, Silha JV, Murphy LJ (2004). Sexual dimorphism and regulation of resistin, adiponectin, and leptin expression in the mouse. *Obes Res* **12**(9): 1481-1491.

Hanisch UK, Rowe W, Sharma S, Meaney MJ, Quirion R (1994). Hypothalamic-pituitary-adrenal activity during chronic central administration of interleukin-2. *Endocrinology* **135**(6): 2465-2472.

Hansson AC, Sommer W, Rimondini R, Andbjer B, Stromberg I, Fuxe K (2003). c-fos reduces corticosterone-mediated effects on neurotrophic factor expression in the rat hippocampal CA1 region. *J Neurosci* **23**(14): 6013-6022.

Hershey T, Black KJ, Hartlein JM, Barch DM, Braver TS, Carl JL*, et al* (2004). Cognitive-pharmacologic functional magnetic resonance imaging in tourette syndrome: a pilot study. *Biol Psychiatry* **55**(9): 916-925.

Hill RA, McInnes KJ, Gong EC, Jones ME, Simpson ER, Boon WC (2007). Estrogen deficient male mice develop compulsive behavior. *Biol Psychiatry* **61**(3): 359-366.

Hoffman KL, Hornig M, Yaddanapudi K, Jabado O, Lipkin WI (2004). A murine model for neuropsychiatric disorders associated with group A beta-hemolytic streptococcal infection. *J Neurosci* **24**(7): 1780-1791.

Horst SA, Linner A, Beineke A, Lehne S, Holtje C, Hecht A*, et al* (2013). Prognostic value and therapeutic potential of TREM-1 in Streptococcus pyogenes- induced sepsis. *J Innate Immun* **5**(6): 581-590.

Hsu LC, Enzler T, Seita J, Timmer AM, Lee CY, Lai TY*, et al* (2011). IL-1beta-driven neutrophilia preserves antibacterial defense in the absence of the kinase IKKbeta. *Nat Immunol* **12**(2): 144-150.

Huber W, Carey VJ, Gentleman R, Anders S, Carlson M, Carvalho BS*, et al* (2015). Orchestrating high-throughput genomic analysis with Bioconductor. *Nat Methods* **12**(2): 115-121.

Hussain D, Cossette MP, Brake WG (2016). High Oestradiol Replacement Reverses Response Memory Bias in Ovariectomised Female Rats Regardless of Dopamine Levels in the Dorsal Striatum. *J Neuroendocrinol* **28**(5).

Igal RA, Wang S, Gonzalez-Baro M, Coleman RA (2001). Mitochondrial glycerol phosphate acyltransferase directs the incorporation of exogenous fatty acids into triacylglycerol. *J Biol Chem* **276**(45): 42205-42212.

Karanth S, Marubayashi U, McCann SM (1992). Influence of dopamine on the altered release of prolactin, luteinizing hormone, and follicle-stimulating hormone induced by interleukin-2 in vitro. *Neuroendocrinology* **56**(6): 871-880.

Karanth S, McCann SM (1991). Anterior pituitary hormone control by interleukin 2. *Proc Natl Acad Sci U S A* **88**(7): 2961-2965.

Kompoliti K, Goetz CG, Leurgans S, Raman R, Comella CL (2001). Estrogen, progesterone, and tic severity in women with Gilles de la Tourette syndrome. *Neurology* **57**(8): 1519.

Krabbe G, Minami SS, Etchegaray JI, Taneja P, Djukic B, Davalos D*, et al* (2017). Microglial NFkappaB-TNFalpha hyperactivation induces obsessive-compulsive behavior in mouse models of progranulin-deficient frontotemporal dementia. *Proc Natl Acad Sci U S A* **114**(19): 5029-5034.

Kurlander RJ (1981). The effects of corticosteroids on IgG Fc receptor and complement receptor-mediated interaction of monocytes with red cells. *Clin Immunol Immunopathol* **20**(3): 325-335.

Lam QL, Zheng BJ, Jin DY, Cao X, Lu L (2007). Leptin induces CD40 expression through the activation of Akt in murine dendritic cells. *J Biol Chem* **282**(38): 27587-27597.

Lawrence T, Bebien M, Liu GY, Nizet V, Karin M (2005). IKKalpha limits macrophage NF-kappaB activation and contributes to the resolution of inflammation. *Nature* **434**(7037): 1138-1143.

Lin CH, Hsieh SC, Keng LT, Lee HS, Chang HT, Liao WY*, et al* (2016). Prospective Evaluation of Procalcitonin, Soluble Triggering Receptor Expressed on Myeloid Cells-1 and C-Reactive Protein in Febrile Patients with Autoimmune Diseases. *PLoS One* **11**(4): e0153938.

Liu X, Herbison AE (2013). Dopamine regulation of gonadotropin-releasing hormone neuron excitability in male and female mice. *Endocrinology* **154**(1): 340-350.

Lord GM, Matarese G, Howard JK, Baker RJ, Bloom SR, Lechler RI (1998). Leptin modulates the T-cell immune response and reverses starvation-induced immunosuppression. *Nature* **394**(6696): 897-901.

Lotan D, Benhar I, Alvarez K, Mascaro-Blanco A, Brimberg L, Frenkel D*, et al* (2014). Behavioral and neural effects of intra-striatal infusion of anti-streptococcal antibodies in rats. *Brain Behav Immun* **38**: 249-262.

Love MI, Huber W, Anders S (2014). Moderated estimation of fold change and dispersion for RNA-seq data with DESeq2. *Genome Biol* **15**(12): 550.

Macrì S, Ceci C, Proietti Onori M, Invernizzi RW, Bartolini E, Altabella L*, et al* (2015). Mice repeatedly exposed to Group-A beta-Haemolytic Streptococcus show perseverative behaviors, impaired sensorimotor gating, and immune activation in rostral diencephalon. *Sci Rep* **5**: 13257.

Macrì S, Pasquali P, Bonsignore LT, Pieretti S, Cirulli F, Chiarotti F*, et al* (2007). Moderate neonatal stress decreases within-group variation in behavioral, immune and HPA responses in adult mice. *PLoS One* **2**(10): e1015.

Magnaghi V, Riva MA, Cavarretta I, Martini L, Melcangi RC (2000). Corticosteroids regulate the gene expression of FGF-1 and FGF-2 in cultured rat astrocytes. *J Mol Neurosci* **15**(1): 11-18.

Martino D, Macerollo A, Leckman JF (2013). Neuroendocrine aspects of Tourette syndrome. *Int Rev Neurobiol* **112**: 239-279.

McCarthy DJ, Chen Y, Smyth GK (2012). Differential expression analysis of multifactor RNA-Seq experiments with respect to biological variation. *Nucleic Acids Res* **40**(10): 4288-4297.

McMurray RW, Ndebele K, Hardy KJ, Jenkins JK (2001). 17-beta-estradiol suppresses IL-2 and IL-2 receptor. *Cytokine* **14**(6): 324-333.

Misso ML, Murata Y, Boon WC, Jones ME, Britt KL, Simpson ER (2003). Cellular and molecular characterization of the adipose phenotype of the aromatase-deficient mouse. *Endocrinology* **144**(4): 1474-1480.

Mitra S, Bastos CP, Bates K, Pereira GS, Bult-Ito A (2016). Ovarian Sex Hormones Modulate Compulsive, Affective and Cognitive Functions in A Non-Induced Mouse Model of Obsessive-Compulsive Disorder. *Front Behav Neurosci* **10**: 215.

Mitsushima D, Takase K, Funabashi T, Kimura F (2008). Gonadal steroid hormones maintain the stress-induced acetylcholine release in the hippocampus: simultaneous measurements of the extracellular acetylcholine and serum corticosterone levels in the same subjects. *Endocrinology* **149**(2): 802-811.

Mocchetti I, Spiga G, Hayes VY, Isackson PJ, Colangelo A (1996). Glucocorticoids differentially increase nerve growth factor and basic fibroblast growth factor expression in the rat brain. *J Neurosci* **16**(6): 2141-2148.

Molteni R, Fumagalli F, Magnaghi V, Roceri M, Gennarelli M, Racagni G*, et al* (2001). Modulation of fibroblast growth factor-2 by stress and corticosteroids: from developmental events to adult brain plasticity. *Brain Res Brain Res Rev* **37**(1-3): 249-258.

Morash B, Johnstone J, Leopold C, Li A, Murphy P, Ur E*, et al* (2000). The regulation of leptin gene expression in the C6 glioblastoma cell line. *Mol Cell Endocrinol* **165**(1-2): 97-105.

Morer A, Chae W, Henegariu O, Bothwell AL, Leckman JF, Kawikova I (2010). Elevated expression of MCP-1, IL-2 and PTPR-N in basal ganglia of Tourette syndrome cases. *Brain Behav Immun* **24**(7): 1069-1073.

Mortensen R, Nissen TN, Blauenfeldt T, Christensen JP, Andersen P, Dietrich J (2015). Adaptive Immunity against Streptococcus pyogenes in Adults Involves Increased IFN-gamma and IgG3 Responses Compared with Children. *J Immunol* **195**(4): 1657-1664.

Murakami Y, Akahoshi T, Aoki N, Toyomoto M, Miyasaka N, Kohsaka H (2009). Intervention of an inflammation amplifier, triggering receptor expressed on myeloid cells 1, for treatment of autoimmune arthritis. *Arthritis Rheum* **60**(6): 1615-1623.

Nomura Y, Segawa M (2003). Neurology of Tourette's syndrome (TS) TS as a developmental dopamine disorder: a hypothesis. *Brain Dev* **25 Suppl 1**: S37-42.

Oliveros JC (2007-2015). Venny. An interactive tool for comparing lists with Venn's diagrams. http://bioinfogp.cnb.csic.es/tools/venny/index.html.

Olivier B, Leahy C, Mullen T, Paylor R, Groppi VE, Sarnyai Z*, et al* (2001). The DBA/2J strain and prepulse inhibition of startle: a model system to test antipsychotics? *Psychopharmacology (Berl)* **156**(2-3): 284-290.

Penza M, Montani C, Romani A, Vignolini P, Pampaloni B, Tanini A*, et al* (2006). Genistein affects adipose tissue deposition in a dose-dependent and gender-specific manner. *Endocrinology* **147**(12): 5740-5751.

Piazza PV, Barrot M, Rouge-Pont F, Marinelli M, Maccari S, Abrous DN*, et al* (1996). Suppression of glucocorticoid secretion and antipsychotic drugs have similar effects on the mesolimbic dopaminergic transmission. *Proc Natl Acad Sci U S A* **93**(26): 15445-15450.

Proietti Onori M, Ceci C, Laviola G, Macrì S (2014). A behavioural test battery to investigate tic-like symptoms, stereotypies, attentional capabilities, and spontaneous locomotion in different mouse strains. *Behav Brain Res* **267**: 95-105.

Rao NP, Venkatasubramanian G, Ravi V, Kalmady S, Cherian A, Yc JR (2015). Plasma cytokine abnormalities in drug-naive, comorbidity-free obsessive-compulsive disorder. *Psychiatry Res* **229**(3): 949-952.

Ren H, Schmalstieg A, van Oers NS, Gaynor RB (2002). I-kappa B kinases alpha and beta have distinct roles in regulating murine T cell function. *J Immunol* **168**(8): 3721-3731.

Ritchie ME, Phipson B, Wu D, Hu Y, Law CW, Shi W*, et al* (2015). limma powers differential expression analyses for RNA-sequencing and microarray studies. *Nucleic Acids Res* **43**(7): e47.

Robinson MD, McCarthy DJ, Smyth GK (2010). edgeR: a Bioconductor package for differential expression analysis of digital gene expression data. *Bioinformatics* **26**(1): 139-140.

Schwabe MJ, Konkol RJ (1992). Menstrual cycle-related fluctuations of tics in Tourette syndrome. *Pediatr Neurol* **8**(1): 43-46.

Schwartz MW, Woods SC, Porte D, Jr., Seeley RJ, Baskin DG (2000). Central nervous system control of food intake. *Nature* **404**(6778): 661-671.

Shams WM, Sanio C, Quinlan MG, Brake WG (2016). 17beta-Estradiol infusions into the dorsal striatum rapidly increase dorsal striatal dopamine release in vivo. *Neuroscience* **330**: 162-170.

Simoncini T, Maffei S, Basta G, Barsacchi G, Genazzani AR, Liao JK*, et al* (2000). Estrogens and glucocorticoids inhibit endothelial vascular cell adhesion molecule-1 expression by different transcriptional mechanisms. *Circ Res* **87**(1): 19-25.

Soneson C, Delorenzi M (2013). A comparison of methods for differential expression analysis of RNA-seq data. *BMC Bioinformatics* **14**: 91.

The UniProt C (2017). UniProt: the universal protein knowledgebase. *Nucleic Acids Res* **45**(D1): D158-D169.

Tsatsaronis JA, Walker MJ, Sanderson-Smith ML (2014). Host responses to group a streptococcus: cell death and inflammation. *PLoS Pathog* **10**(8): e1004266.

Turgeon JL, Waring DW (1999). Androgen modulation of luteinizing hormone secretion by female rat gonadotropes. *Endocrinology* **140**(4): 1767-1774.

Turzillo AM, Nolan TE, Nett TM (1998). Regulation of gonadotropin-releasing hormone (GnRH) receptor gene expression in sheep: interaction of GnRH and estradiol. *Endocrinology* **139**(12): 4890-4894.

Van den Buuse M, Eikelis N (2001). Estrogen increases prepulse inhibition of acoustic startle in rats. *Eur J Pharmacol* **425**(1): 33-41.

van den Buuse M, Simpson ER, Jones ME (2003). Prepulse inhibition of acoustic startle in aromatase knock-out mice: effects of age and gender. *Genes Brain Behav* **2**(2): 93-102.

Vandermoere F, El Yazidi-Belkoura I, Adriaenssens E, Lemoine J, Hondermarck H (2005). The antiapoptotic effect of fibroblast growth factor-2 is mediated through nuclear factor-kappaB activation induced via interaction between Akt and IkappaB kinase-beta in breast cancer cells. *Oncogene* **24**(35): 5482-5491.

Wang L, Wang S, Li W (2012). RSeQC: quality control of RNA-seq experiments. *Bioinformatics* **28**(16): 2184-2185.

Wolf WA, Martin JL, Kartje GL, Farrer RG (2014). Evidence for fibroblast growth factor-2 as a mediator of amphetamine-enhanced motor improvement following stroke. *PLoS One* **9**(9): e108031.

Wu TD, Nacu S (2010). Fast and SNP-tolerant detection of complex variants and splicing in short reads. *Bioinformatics* **26**(7): 873-881.

Yaddanapudi K, Hornig M, Serge R, De Miranda J, Baghban A, Villar G*, et al* (2010). Passive transfer of streptococcus-induced antibodies reproduces behavioral disturbances in a mouse model of pediatric autoimmune neuropsychiatric disorders associated with streptococcal infection. *Mol Psychiatry* **15**(7): 712-726.

Zhang ZH, Jhaveri DJ, Marshall VM, Bauer DC, Edson J, Narayanan RK*, et al* (2014). A comparative study of techniques for differential expression analysis on RNA-Seq data. *PLoS One* **9**(8): e103207.

Zhong J, Huang W, Deng Q, Wu M, Jiang H, Lin X*, et al* (2016). Inhibition of TREM-1 and Dectin-1 Alleviates the Severity of Fungal Keratitis by Modulating Innate Immune Responses. *PLoS One* **11**(3): e0150114.

Zoratto F, Berry A, Anzidei F, Fiore M, Alleva E, Laviola G*, et al* (2011). Effects of maternal L-tryptophan depletion and corticosterone administration on neurobehavioral adjustments in mouse dams and their adolescent and adult daughters. *Prog Neuropsychopharmacol Biol Psychiatry* **35**(6): 1479-1492.

Zoratto F, Fiore M, Ali SF, Laviola G, Macrì S (2013). Neonatal tryptophan depletion and corticosterone supplementation modify emotional responses in adult male mice. *Psychoneuroendocrinology* **38**(1): 24-39.
